# Refining Lung Cancer Care: The Emerging Role of Liquid Biopsy in Screening, Diagnosis, and Precision Oncology

**DOI:** 10.3390/cancers18193140

**Published:** 2026-09-28

**Authors:** Hicham Mansour, Omayma Mazouji, Ayman El Hammouti, Ihasane Alloubi, Roberto Incitti, Saïd El Kazzouli, Abdelhak Ouhajjou, Chakib Nejjari

**Affiliations:** 1 Euromed Research Center, Euromed University of Fes (UEMF), Fes 30000, Morocco; r.incitti@ueuromed.org (R.I.); s.elkazzouli@ueuromed.org (S.E.K.);; 2 Laboratory of Molecular Chemistry, Materials and Environment (LCME), Department of Geology, Environment and Health (GES), Faculty of Applied Sciences (FSA), Mohamed 1st University, Nador 62700, Morocco; mazouji.omayma@ump.ac.ma (O.M.); ayman.elhammouti.2016@gmail.com (A.E.H.); 3 Thoracic Surgery Department, Mohamed VI University Hospital Center, Tangier 90000, Morocco; i.alloubi@uae.ac.ma; 4 Al Azhar Oncology Center, Rabat 10000, Morocco; ouhajjou@alazharoncologie.com; 5 Faculty of Medicine, Pharmacy and Dentistry, Sidi Mohamed Ben Abdellah University, Fes 30070, Morocco

**Keywords:** lung cancer, blood test, screening, diagnosis, follow up, treatment guidance, non-invasive, genetic test, epigenetic test, liquid biopsy

## Abstract

**Simple Summary:**

Lung cancer remains the leading cause of cancer-related mortality worldwide, highlighting the urgent need for more effective strategies for early detection. Timely diagnosis improves treatment options, patient survival, and quality of life while reducing healthcare costs. Although low-dose computed tomography is currently the standard screening method for high-risk individuals, its clinical performance is limited by false-positive findings and restricted accessibility. Emerging biomarker-based liquid biopsy offers a promising non-invasive approach for cancer detection, disease monitoring, and treatment selection. Continued analytical, clinical, and regulatory advances are expected to facilitate their integration into routine clinical practice as complementary tools to tissue biopsy and LDCT screening technology and, in some cases, as an alternative approach.

**Abstract:**

Lung cancer (LC) remains the leading cause of cancer-related mortality worldwide, representing a major public health challenge. Although low-dose computed tomography is currently the standard screening method for high-risk individuals to reduce mortality, its clinical performance is limited by false-positive findings and restricted accessibility. Emerging biomarker-based liquid biopsy offers a promising non-invasive approach for cancer detection. This narrative review provides a critical overview and evaluation of clinically endorsed liquid biopsy applications for guiding treatment in advanced-stage lung cancer, as well as investigational advances in liquid biopsy for early cancer detection. We also address challenges of current liquid biopsy technologies, the integration of artificial intelligence, implementation improvements, and future perspectives for their adoption into routine clinical practice. To complement this narrative review, a structured search of the ClinicalTrials.gov registry was performed to identify clinical trials evaluating liquid biopsy in lung cancer. Current evidence supports the clinical use of liquid biopsy for molecular profiling and treatment selection in advanced non-small cell lung cancer (NSCLC), whereas its application in early detection, screening, and minimal residual disease (MRD)-guided management remains investigational and requires further prospective validation. Overall, liquid biopsy represents a promising complementary approach to advance precision oncology and improve lung cancer management.

## 1. Introduction

To date, lung cancer (LC) remains the leading cause of cancer-related death worldwide in both men and women and is among the most commonly diagnosed cancers globally. It remains a major public health challenge and a substantial economic burden. According to the International Agency for Research on Cancer (IARC) GLOBOCAN 2024 estimates, lung cancer remains the most commonly diagnosed cancer and the leading cause of cancer-related mortality worldwide, with approximately 2.64 million new cases and 1.86 million deaths in 2024. Asia bears the highest disease burden, accounting for nearly two-thirds of both incident cases and deaths, followed by Europe and North America [1].

The 5-year survival rate for LC diagnosed at stage I varies by histological subtype, reaching approximately 67% for non-small cell lung cancer (NSCLC) and 34% for small cell lung cancer (SCLC). In contrast, for patients diagnosed at stage IV or with metastatic disease, 5-year survival rates decline sharply to around 12% and 4% for NSCLC and SCLC, respectively [2]. Although comparable stage-dependent survival patterns are observed in other malignancies, LC continues to cause more deaths than other high-incidence cancers combined, including breast and colorectal cancers. These data underscore the pivotal role of early detection in reducing lung cancer-related mortality.

Lung cancer (LC) screening targets individuals at elevated risk of developing the disease with the objective of identifying tumors at an early stage, when curative treatment is more likely to be effective. Early diagnosis not only improves patients’ chances of successful treatment and survival but also helps reduce healthcare costs, as managing advanced-stage disease is substantially more expensive than treating early-stage cancer. Consequently, many countries have established organized screening programs for eligible high-risk populations to decrease LC-related mortality and improve quality of life. Among the eligibility criteria, cigarette smoking remains the most important risk factor and is the primary determinant for selecting individuals for LC screening [3].

Several professional organizations and expert panels recommend specific approaches for lung cancer (LC) screening. Among these, low-dose computed tomography (LDCT) is currently regarded as the reference screening modality and is recommended by the U.S. Preventive Services Task Force (USPSTF) for eligible high-risk individuals. Despite its widespread adoption and demonstrated effectiveness in detecting early-stage lung cancer, LDCT has important limitations, including a relatively high rate of false-positive findings and limited detection efficiency in some cases. Consequently, the overall balance between its clinical benefits and potential drawbacks continues to be actively evaluated and discussed within the scientific community [4].

Historically, several other screening approaches, including chest X-ray and sputum cytology, were investigated for the early detection of lung cancer. However, these methods failed to demonstrate a sufficient reduction in lung cancer mortality and are no longer recommended for routine screening. Hopefully, emerging biomarker-based liquid biopsy tests offer a promising non-invasive approach for early-stage cancer detection. These tests are under development and present huge potential for LC screening—for example, the multi-cancer early detection (MCED) test developed by the Grail company.

Beyond screening, different clinical objectives in lung cancer management require distinct diagnostic strategies. While histopathological examination remains the gold standard for diagnosis, molecular profiling has become essential for treatment selection, and additional tools are increasingly being explored for minimal residual disease detection, recurrence surveillance, and treatment-response monitoring [4,5]. In this context, liquid biopsy has emerged as a promising complementary approach for improving lung cancer management through minimally invasive molecular analysis. Although its current clinical application is primarily limited to molecular profiling and treatment selection in advanced non-small cell lung cancer, ongoing research is expanding its potential role in early detection, disease monitoring, minimal residual disease assessment, and treatment personalization.

A comprehensive literature search was performed using the PubMed and ClinicalTrials.gov databases to identify relevant studies addressing liquid biopsy and its applications in lung cancer. This review provides a comprehensive overview of the current evidence on liquid biopsy in lung cancer, covering its role in screening, diagnostic integration with conventional approaches, molecular profiling, therapeutic monitoring, and precision oncology. In addition, we critically discuss the strengths and limitations of current liquid biopsy technologies, recent technological advances, the integration of artificial intelligence, regulatory and implementation challenges, and future perspectives for their integration into routine clinical practice.

## 2. Methodology

This article was prepared as a narrative review of the current evidence on the role of liquid biopsy in lung cancer screening, diagnosis, monitoring, and precision oncology. For the narrative literature review, a literature search was performed using the PubMed database to identify primary studies, clinical trials, systematic and narrative reviews, and relevant clinical practice guidelines addressing liquid biopsy and its applications in lung cancer. The search covered publications indexed in PubMed from 1968 through 2025. A comprehensive literature search was performed using the PubMed database to identify relevant publications. The search included combinations of the following keywords: “lung cancer,” “liquid biopsy,” “screening,” “diagnosis,” and “lung cancer management”. Original research articles, review articles, clinical trials, and relevant clinical practice guidelines published in English were considered based on their scientific relevance and contribution to the topic. Publications were excluded if they were conference abstracts, editorials, letters, commentaries, case reports, or studies not involving human subjects. Studies that were not related to lung cancer were also excluded.

To complement the narrative research review, a structured literature search of the ClinicalTrials.gov registry was performed to identify clinical trials evaluating the application of liquid biopsy in lung cancer. The registry was searched using the predefined keywords “lung cancer” and “liquid biopsy.” Eligible studies were required to have a study start date on or after 1 January 2015. Trials that started before 1 January 2015 were excluded. Regarding study completion, trials with a primary completion date before 1 January 2022 were excluded, whereas trials with a primary completion date on or after 1 January 2022, including trials with an estimated completion date after 2022, were eligible for inclusion. Therefore, the search included ongoing trials and trials with future estimated completion dates. Trials evaluating liquid biopsy approaches for lung cancer diagnosis, molecular profiling, treatment selection, therapeutic monitoring, minimal residual disease, or disease surveillance were included. Studies not related to lung cancer or not involving liquid biopsy technologies were excluded.

For each eligible study, the following information was extracted: National Clinical Trial (NCT) identifier, study title, study design, study phase, number of study locations, tumor subtype, disease stage, number of participants, liquid biopsy analyte (e.g., circulating tumor DNA, circulating tumor cells, extracellular vesicles, or other biomarkers), test type (e.g., genetic or epigenetic) intended clinical application, approach used to generate the data, gene tested, tissue concordance if applicable, primary outcome if available, starting date and research completion or Estimation of the completing date. A total of 166 clinical trials met the eligibility criteria and were included in the descriptive analysis. The registry data were summarized to provide an overview of current research directions and the clinical development of liquid biopsy technologies in lung cancer.

## 3. Results and Discussion

### 3.1. Gold Standard Lung Cancer Screening Tools

Several tools have been evaluated in clinical trials to screen for lung cancer, like chest radiographs (CXR), sputum cytology and LDCT [6,7,8]. Among these tools, LDCT is the only tool that has demonstrated a significant reduction in lung cancer-specific mortality and is therefore recommended for its performance in detecting small, early-stage lung cancers. LDCT is a non-contrast computed tomography imaging technique that uses low-dose X-ray radiation to generate high-resolution cross-sectional images of the lungs during a single breath-hold. The radiation exposure associated with LDCT is substantially lower than that of conventional diagnostic chest CT, with a typical effective dose of approximately 1–1.5 mSv per scan, although the actual dose varies according to scanner technology, acquisition protocol, and patient characteristics [9,10]. Artificial intelligence may be used as a complementary tool to assist image interpretation, pulmonary nodule detection, and risk stratification. A series of detailed images of areas inside the lung are taken from different angles and are used to generate three-dimensional views of the target organ to detect pulmonary nodules. The LDCT using a multi-detector scanner has high sensitivity and is able to detect small lung cancer nodules [9,11]. To standardize the interpretation of screening examinations and subsequent management, the American College of Radiology developed the Lung CT Screening Reporting and Data System (Lung-RADS). Lung-RADS categorizes screening findings according to the estimated likelihood of malignancy and links these categories to standardized management recommendations, including routine annual screening, short-interval CT follow-up, or diagnostic evaluation [12]. Despite its high sensitivity, LDCT has relatively low specificity, resulting in a substantial number of false-positive findings that often require additional imaging or invasive diagnostic procedures. The use of structured reporting and risk-stratification systems such as Lung-RADS therefore helps reduce unnecessary follow-up procedures while providing a standardized framework for determining the appropriate interval and intensity of follow-up. Therefore, while LDCT represents the standard imaging modality for lung cancer screening in high-risk populations, its clinical implementation should be accompanied by appropriate risk stratification and follow-up strategies to minimize unnecessary interventions and patient anxiety.

### 3.2. Major Lung Cancer Screening Programs

In many countries, several clinical trials have evaluated the effectiveness of LDCT as a screening tool for reducing LC mortality and avoiding harm to unaffected individuals from over-diagnosis [9]. Several countries are organizing screening programs using LDCT and have shown a reduction in mortality through these screening programs. Landmark studies, including the National Lung Screening Trial (NLST) and the NELSON trial, demonstrated significant reductions in lung cancer mortality, leading to the implementation of LDCT screening programs in several countries [9,13,14,15,16]. The principal characteristics and outcomes of these trials are summarized in Table 1.

These findings strongly support the importance of LC screening and the effectiveness of LDCT in this clinical setting. Currently, several medical societies, including USPSTF, ACCP, ACS, CCO, AATS, and others, recommend annual LC screening with LDCT. Specifically, annual screening is advised for individuals aged 50–80 years with a 20 pack-year smoking history who currently smoke or have quit within the past 15 years [16,17].

LDCT has become the reference imaging modality for lung cancer (LC) screening and has demonstrated its ability to reduce disease-specific mortality in high-risk populations. Despite its established clinical value, several limitations have prompted ongoing efforts to improve current screening strategies and develop complementary approaches. Although LDCT remains the only screening modality proven to reduce lung cancer mortality, its overall clinical effectiveness depends not only on its diagnostic performance but also on the appropriate selection of high-risk individuals and adherence to screening protocols. Consequently, its benefits may not be fully realized in broader populations outside the current screening recommendations. The diagnostic performance of LDCT is influenced by multiple factors, including patient characteristics (e.g., age and ethnicity), tumor size and location, lung parenchymal density, the interval between screening examinations, and the quality and interpretation of the acquired images [16]. These sources of variability can affect the reproducibility of screening outcomes across different healthcare settings and emphasize the need for standardized protocols and quality assurance measures. In addition, LDCT screening is associated with several well-recognized challenges. These include over-diagnosis, which may expose patients to unnecessary interventions, a substantial number of false-positive findings that may require additional imaging or invasive diagnostic procedures, and false-negative results, particularly for small or difficult-to-detect lesions. These limitations not only increase healthcare costs but may also negatively affect patients through procedure-related complications, psychological distress, and reduced confidence in screening programs. Furthermore, image interpretation is time-consuming and highly dependent on radiologist expertise, contributing to inter-observer variability. Another limitation is that LDCT provides limited information regarding disease recurrence or treatment response. Although the radiation dose is considerably lower than that of conventional CT, repeated examinations still result in cumulative radiation exposure, which may reduce participation in long-term screening programs. Finally, the acquisition, maintenance, and operation of LDCT equipment require significant financial investment, potentially limiting access to screening in resource-constrained healthcare settings [18]. Taken together, these limitations suggest that, despite its central role in lung cancer screening, LDCT alone is insufficient to address all clinical needs. This has driven growing interest in combining LDCT with minimally invasive approaches, such as liquid biopsy or volatile organic compound (VOC) assays, which may improve detection specificity, facilitate disease monitoring, and ultimately enhance the overall effectiveness of lung cancer screening and management. VOC approaches analyze exhaled air rather than blood-derived or other circulating biological materials. Published studies have reported promising diagnostic performance for VOC-based breath analysis, but substantial heterogeneity in breath collection, environmental conditions, analytical platforms, and normalization procedures remains a major barrier to clinical implementation [19,20]. Because the primary focus of our review is liquid biopsy, VOC and exhaled-breath assays are outside the strict scope of liquid biopsy (Figure 1).

### 3.3. Liquid Biopsy Tests Endorsed or Under Investigation for Lung Cancer Detection, Follow-Up and Treatment Guidance

#### 3.3.1. Liquid Biopsy Tests for Lung Cancer Screening

To date, no liquid biopsy test has been officially endorsed as a stand-alone test for lung cancer (LC) screening. However, several assays are currently being investigated, and some are commercially available in certain markets. Among these, the Galleri™ multi-cancer early detection (MCED) test, developed by GRAIL, is one of the most advanced assays and has been evaluated in prospective clinical studies, including the PATHFINDER study. Nevertheless, its use for population-based cancer screening, and particularly as a replacement for LDCT in lung cancer screening, remains investigational. The test uses next-generation sequencing (NGS) of cell-free DNA (cfDNA) shed by tumors into the bloodstream and detects cancer-associated DNA methylation patterns to identify a cancer signal and predict its tissue of origin [21]. In the published PATHFINDER clinical study, the test was assessed in a cohort of average-risk, asymptomatic outpatient population aged 50 and older without any clinical symptoms of cancer [22]. Among the 6621 recruited participants, a “cancer signal detected” result was reported in 92 participants (1.4%), while no cancer signal was detected in 6529 participants (98.6%). Following diagnostic evaluation, cancer was confirmed in 35 of the 92 participants with a cancer signal detected result, corresponding to a positive predictive value (PPV) of 38%. The MCED test demonstrated a specificity of approximately 99%. This advanced assay combines a genetic test with an AI algorithm to improve data quality and diagnostic accuracy, effectively detecting both solid tumors such as LC and hematological cancers. The test also predicts the cancer signal origin (CSO). Notably, nearly half of the newly diagnosed cancers (48%) were detected at an early stage, highlighting the potential impact on early cancer detection [22]. Despite its promising performance, the GRAIL Galleri™ multi-cancer early detection test has several important limitations that currently preclude its use as a stand-alone screening tool for lung cancer. Its sensitivity remains limited for early-stage disease because small tumors often release insufficient amounts of circulating tumor DNA into the bloodstream. Although the assay demonstrates high specificity, false-positive and false-negative results may occur, necessitating additional diagnostic investigations and potentially increasing patient anxiety and healthcare costs. Furthermore, a positive MCED result is not diagnostic and requires confirmation through conventional imaging and/or tissue biopsy. Although Galleri™ is commercially available in some markets, its clinical utility as a population-level cancer screening test remains under evaluation. In addition, the test has not received FDA approval as a screening assay, is associated with relatively high costs and limited reimbursement, and is not currently recommended by major clinical guidelines as a replacement for established screening modalities such as LDCT. Therefore, MCED assays should presently be considered complementary tools whose routine implementation will require further prospective validation, demonstration of clinical utility, and cost-effectiveness [23].

Although the investigation of ctDNA in liquid biopsy, particularly MCED tests, have shown considerable promise for lung cancer detection, several other biomarkers have also been evaluated, including circulating tumor cells (CTCs), exosomes, and microRNAs (miRNAs). Each biomarker presents distinct advantages and limitations with respect to analytical performance, clinical applicability, and implementation. A comparative overview is provided in Table 2 and Figure 2.

As summarized in Table 2, ctDNA currently represents the most clinically used liquid biopsy biomarker, with four FDA-approved assays available for molecular profiling in advanced NSCLC, including Agilent Resolution ctDx FIRST assay, Cobas EGFR Mutation Test v2, FoundationOne Liquid CDx, and Guardant360 CDx (Table 3). However, its sensitivity remains limited in early-stage disease due to the low abundance of ctDNA. In contrast, CTCs provide intact tumor cells for comprehensive molecular characterization but are technically challenging to isolate because of their rarity. Exosomes and circulating miRNAs markers offer complementary biological information and may improve early detection, although their widespread clinical implementation is currently limited by the lack of standardized analytical methods and prospective validation. These findings suggest that no single biomarker adequately addresses all clinical needs, and future screening strategies will likely rely on multimodal approaches integrating molecular biomarkers with imaging modalities such as LDCT [24,25,26,27,28,29,30,31].

#### 3.3.2. Liquid Biopsy Tests Endorsed for Other Lung Cancer Settings

**FDA-approved liquid biopsy assays:** In clinical practice, we already have several non-invasive tools already that are endorsed and used in other lung cancer settings. These tests are used mainly in patient selection for treatment, to improve patient survival and quality of life. These tests are based on the genotyping of circulating tumor DNA released by the tumor. These tests are considered in clinical settings when rapid results are needed and tissue is unavailable or when used to complement tissue data. The ctDNA assays can be used to genotype advanced cancers and select patients for targeted therapies, and when considering a treatment for which there are specific genomic biomarker-based contraindications or exclusions [32,33]. The clinically relevant biomarkers detected by FDA-authorized liquid biopsy assays and their corresponding targeted therapies are summarized in Table 3. As shown, several plasma-based companion diagnostic assays have received FDA approval for detecting clinically actionable alterations in NSCLC, including EGFR, ALK, ROS1, BRAF V600E, MET exon 14 skipping, KRAS G12C, ERBB2 (HER2) and NTRK1-3, thereby facilitating the selection of patients eligible for targeted therapies.

**Liquid biopsy in personalized treatment:** Liquid biopsy plays an increasingly important role in personalized treatment of advanced NSCLC and is recommended as a complementary approach to tissue genotyping in selected clinical situations. Plasma ctDNA testing is particularly useful when tumor tissue is unavailable, insufficient for comprehensive molecular analysis, or when rapid identification of actionable genomic alterations is required to initiate targeted therapy. Because the specificity of plasma ctDNA assays is high, detection of a clinically actionable alteration can directly guide treatment selection. However, a negative plasma result does not exclude the presence of a targetable genomic alteration, particularly in patients with low tumor DNA shedding. Therefore, current international guidelines recommend reflex tissue molecular testing whenever plasma testing is negative and adequate tissue is available. Beyond initial molecular profiling, serial liquid biopsy enables non-invasive monitoring of treatment response and the identification of acquired resistance mechanisms during targeted therapy [34,35].

**Liquid biopsy for detecting resistance mechanisms:** Beyond baseline molecular profiling, liquid biopsy has become an important tool for identifying acquired resistance mechanisms during targeted therapy. Serial ctDNA analysis enables non-invasive monitoring of tumor evolution and the emergence of resistance-associated genomic alterations before radiological disease progression becomes evident. Detection of resistance mutations allows timely modification of treatment strategies and selection of next-line targeted therapies. Examples include the EGFR T790M mutation, which predicts resistance to first- and second-generation EGFR tyrosine kinase inhibitors and identifies patients eligible for Osimertinib, as well as secondary alterations involving MET amplification, HER2 amplification, KRAS mutations, ALK kinase-domain mutations, and other bypass signaling pathways that contribute to therapeutic resistance. Similarly, in tumors harboring NTRK1, NTRK2, or NTRK3 fusions, serial liquid biopsy can identify acquired on-target resistance mutations following treatment with first-generation TRK inhibitors such as larotrectinib or entrectinib. These alterations include solvent-front mutations such as NTRK1 G595R, NTRK2 G639L/G639R, and NTRK3 G623R, as well as gatekeeper and xDFG mutations affecting the respective NTRK kinase domains. Importantly, NTRK1 and NTRK3 resistance mutations have been detected in plasma ctDNA at disease progression, demonstrating the potential value of liquid biopsy for longitudinal monitoring of TRK inhibitor resistance and guiding subsequent treatment selection [12,20,35,36,37,38,39,40]. Although tissue biopsy remains necessary in selected cases, liquid biopsy provides a rapid and minimally invasive approach for longitudinal molecular monitoring and is increasingly incorporated into routine management of advanced NSCLC [36,37].

**Liquid biopsy for detecting minimal residual disease (MRD) and longitudinal monitoring:** Minimal residual disease (MRD) and longitudinal monitoring represent emerging applications of liquid biopsy in lung cancer management. Following curative-intent surgery or definitive chemoradiotherapy, the detection of ctDNA may reveal molecular residual disease before clinical or radiological evidence of recurrence becomes apparent. Numerous studies have demonstrated that postoperative ctDNA positivity is strongly associated with an increased risk of disease recurrence and shorter disease-free survival, whereas patients with undetectable ctDNA generally experience more favorable outcomes. During systemic therapy, serial ctDNA analysis enables real-time assessment of treatment response by monitoring changes in ctDNA levels over time. A decline or clearance of ctDNA is generally associated with therapeutic response, whereas persistent or increasing ctDNA levels may indicate treatment resistance or disease progression. Importantly, liquid biopsy should be interpreted alongside conventional imaging, as these approaches provide complementary information regarding tumor burden and disease evolution. Although substantial evidence supports the clinical validity of ctDNA as a prognostic biomarker for MRD detection and recurrence risk assessment, its clinical utility, defined as the demonstration that ctDNA-guided treatment decisions improve patient outcomes, has not yet been conclusively established. Consequently, prospective randomized clinical trials are still required before ctDNA-guided MRD assessment and longitudinal monitoring can be routinely incorporated into standard lung cancer management [34].

#### 3.3.3. Clinical Trials Landscape of Liquid Biopsy Tests in Lung Cancer

The clinical utility of liquid biopsy in lung cancer is rapidly expanding beyond molecular characterization toward real-time disease management and personalized treatment strategies. The clinical trials summarized in Supplementary Table S1 provide a comprehensive overview of the current clinical development, validation, and implementation status of liquid biopsy approaches in lung cancer. This overview highlights the translation of these technologies into clinical practice by summarizing prospective studies evaluating their clinical utility in the real world.

A comprehensive analysis of the clinical trials included in this review identified approximately 166 registered clinical studies investigating the application of liquid biopsy in lung cancer. These studies were initiated between 2015 and 2025, with several ongoing trials expected to continue until 2035, reflecting the rapidly expanding interest in liquid biopsy technologies within thoracic oncology. The increasing number of registered trials over the past decade highlights the growing clinical acceptance of liquid biopsy as a promising non-invasive tool for cancer diagnosis, molecular profiling, treatment selection, and disease monitoring.

**Regarding tumor type**, non-small cell lung cancer (NSCLC) represented the most frequently investigated malignancies, accounting for approximately 80–85% of all studies. This predominance reflects the high global incidence of NSCLC and its well-established dependence on molecular biomarkers for therapeutic decision-making. This predominance is consistent with the epidemiology of lung cancer, as NSCLC accounts for approximately 85% of all lung cancer diagnoses worldwide [41,42]. A smaller proportion of studies included patients with lung cancer regardless of histological subtype, while only a limited number focused on small cell lung cancer (SCLC), or lung neuroendocrine tumors.

**Analysis of disease stage** showed that most clinical trials enrolled patients with advanced or metastatic disease, particularly those with stage III, stage IIIB, and stage IV. Advanced-stage patients remain the principal target population because they frequently require comprehensive molecular profiling to identify actionable genomic alterations and guide targeted therapies. However, an increasing number of studies now investigate liquid biopsy in early-stage disease, particularly minimal residual disease (MRD) detection, postoperative surveillance, recurrence monitoring, and lung cancer screening among high-risk populations. This clinical expansion illustrates the expanding role of liquid biopsy beyond advanced disease management toward earlier stages of cancer detection and intervention [43].

**Among the various circulating biomarkers** investigated, circulating tumor DNA (ctDNA) was by far the most frequently studied analyte, representing the cornerstone of current liquid biopsy research. Several studies have supported the view that CtDNA has become the most informative tumor-derived material for most types of cancer, including non-small cell lung cancer [44,45]. CtDNA was primarily employed for comprehensive genomic profiling. Other analytes, including circulating tumor cells (CTCs), exosomes, cell-free RNA (cfRNA), circulating tumor RNA (ctRNA), and microRNAs (miRNAs), were investigated less frequently but demonstrated increasing utilization in more recent studies. Exosome-based biomarkers and epigenetic markers have gained considerable attention as promising tools for early cancer detection and multi-omics biomarker development.

**The clinical applications** of liquid biopsy varied considerably across the included studies. Treatment guidance represented the most common clinical objective, accounting for approximately one-third of all clinical trials. These studies evaluated the ability of liquid biopsy to identify actionable mutations, guide targeted therapy selection, monitor therapeutic response, detect resistance mutations, and optimize personalized treatment strategies. Recent evidence further demonstrates that serial ctDNA analysis can predict treatment response, detect molecular progression several months before radiological progression, and identify patients with minimal residual disease who are most likely to benefit from adjuvant therapy, thereby supporting more individualized treatment decisions [46,47,48,49,50,51]. Diagnosis constituted another major application, with numerous clinical trials assessing the ability of liquid biopsy to shorten diagnostic pathways, reduce dependence on invasive tissue biopsies, and improve molecular diagnosis. Other frequently investigated clinical purposes included follow-up monitoring, screening and overall cancer management.

**Analysis of the approaches** used demonstrated that next-generation sequencing (NGS) was the predominant analytical platform employed throughout the clinical trials, reflecting its central role in comprehensive genomic profiling and the simultaneous detection of multiple actionable genomic alterations in lung cancer [52,53]. Among NGS applications, targeted sequencing, whole-exome, whole-genome, and methylation sequencing are the most widely used. Digital droplet PCR (ddPCR) represents the second most frequently utilized technique, because of its high sensitivity for detecting low frequency variants and low cost. Additional technologies used include real-time PCR or conventional PCR. Several of the most recent studies have also incorporated artificial intelligence and machine learning algorithms to improve biomarker interpretation and clinical decision-making.

**The molecular targets** evaluated reflected the current landscape of precision oncology in lung cancer, as illustrated in Figure 3. EGFR was the most frequently analyzed biomarker across the included studies and the most studied gene [54,55], consistent with its central role in guiding targeted therapies for NSCLC. Other actionable alterations such as EGFR, ALK, ROS1, BRAF, MET, RET, and NTRK are routinely assessed to guide targeted therapies. Beyond initial molecular diagnosis, liquid biopsy has become an important tool for identifying resistance mechanisms during targeted therapy, including the detection of EGFR T790M and other acquired resistance alterations, enabling timely modification of treatment strategies without the need for repeated invasive tissue biopsies [34,56]. The frequent assessment of these biomarkers emphasizes the growing importance of comprehensive molecular profiling for identifying actionable genomic alterations and predicting treatment responses.

In conclusion, based on study design, most of the included clinical trials were observational. Interventional studies represented a substantial proportion of the dataset, whereas randomized controlled trials were comparatively less common. Among interventional investigations, Phase II studies were the most frequently reported, followed by phase III trials, while relatively few early-phase studies were identified. This distribution suggests that liquid biopsy technologies have progressed, reflecting the current emphasis on clinical feasibility, and real-world implementation of liquid biopsy technologies. Overall, the analysis demonstrates a clear evolution in the clinical applications of liquid biopsy over the past decade. These findings indicate that liquid biopsy has evolved from an adjunct molecular diagnostic technique into a comprehensive precision medicine platform capable of supporting diagnosis, therapeutic decision-making, disease monitoring, and personalized patient management across the entire continuum of lung cancer care.

#### 3.3.4. Liquid Biopsy Challenges

Despite its considerable clinical potential, liquid biopsy still faces several technical, clinical, and interpretative challenges that must be overcome to facilitate its widespread integration into routine clinical practice for lung cancer screening, diagnosis, treatment selection, and patient monitoring. These limitations include:

**Liquid biopsy utility information dissemination:** When pursuing diagnosis or treatment follow-up, if issues arise with tissue biopsy such as unavailability or inaccessibility, liquid biopsy is not systematically considered for all cases, even though it could provide valuable information for diagnosis, treatment guidance and disease monitoring.

The overall dissemination of information about the advantages and the added value of liquid biopsy and its biomarkers in lung cancer management remains, unfortunately, a limiting factor. Much more effort is needed to raise awareness about the added value of liquid biopsy, both as a complement to tissue biopsy and as an alternative when tissue is unavailable.

**Tissue biopsy markers: translation to liquid biopsy:** Unfortunately, to date, not all biomarkers routinely assessed in tissue samples can be reliably evaluated using liquid biopsy. One major limitation is that ctDNA analysis primarily provides information at the DNA level, whereas tissue samples also allow the assessment of proteins. In addition, only a fraction of the tumor genome is released into the bloodstream, resulting in limited representation of the tumor’s molecular landscape. Moreover, ctDNA is highly fragmented, which may further complicate the reliable detection and the characterization of certain alterations. In particular, PD-L1 expression remains a tissue-based biomarker assessed by immunohistochemistry, while tumor mutational burden (TMB) is preferentially evaluated using tissue, although blood-based TMB assays are still under clinical investigation and have not been widely adopted in routine practice. Similarly, microsatellite instability-high (MSI-H) is an established predictive biomarker for response to immune checkpoint inhibitors. Although MSI-H is relatively uncommon in lung cancer, it has been identified in both non-small cell lung cancer and small cell lung cancer. Several biomarkers assessed in tissue have clinical utility for treatment selection in lung cancer, including PD-L1 expression, TMB-H, and MSI-H. PD-L1 expression is routinely used to guide immunotherapy selection in advanced NSCLC; for example, pembrolizumab monotherapy is an established first-line option for patients with metastatic NSCLC whose tumors have high PD-L1 expression (TPS ≥ 50%) and no EGFR or ALK genomic alterations. TMB-H (≥10 mutations/Mb) and MSI-H/dMMR also have tumor-agnostic clinical utility for selecting patients for pembrolizumab after progression following prior treatment when no satisfactory alternative treatment options are available [57]. Importantly, emerging evidence indicates that MSI status can also be assessed using blood-based next-generation sequencing approaches, supporting the potential role of liquid biopsy for MSI assessment. However, because MSI-H remains an uncommon biomarker in lung cancer and blood-based MSI assessment requires further validation [39], its clinical use remains limited.

**Technical challenges in taking samples for genetic testing:** In some cases, we have inadequate liquid biopsy samples for biomarker testing. Some samples contain no tumor-derived material or only minimal traces, which makes it difficult to assess gene mutations or methylation, particularly somatic mutations present at low frequency. To overcome this issue, several blood draws performed at different time points should be considered. In such cases, DNA extraction should be prioritized first, before proceeding to genetic alteration testing, in order to increase the likelihood of successful detection and avoid wasting time and consumables on unsuccessful testing.

**Pre-analytical considerations:** The accuracy of liquid biopsy results is highly dependent on appropriate pre-analytical handling. Plasma is generally preferred over serum because serum contains higher levels of contaminating genomic DNA released from lysed blood cells, which may reduce assay sensitivity. In addition, the use of specialized blood collection tubes and prompt sample processing is essential to preserve circulating cell-free DNA integrity and minimize leukocyte lysis. Standardized protocols for sample collection, storage, transportation, and processing are therefore critical to ensure reliable and reproducible molecular analyses.

**Tumor fraction and assay sensitivity:** The proportion of tumor-derived DNA within the total circulating cell-free DNA (tumor fraction) is often extremely low, especially in early-stage lung cancer. Consequently, highly sensitive analytical platforms with very low limits of detection are required to identify low-frequency mutations accurately. Insufficient tumor fraction may lead to false-negative results and should therefore be considered when interpreting negative liquid biopsy findings.

**False-positive results and clonal hematopoiesis:** Although liquid biopsy demonstrates high analytical specificity, false-positive results may occur because of technical artifacts or clonal hematopoiesis of indeterminate potential (CHIP). CHIP refers to age-related somatic mutations and methylation arising from hematopoietic stem cells rather than the tumor itself. These DNA abnormalities may be mistakenly interpreted as tumor-derived alterations if appropriate bioinformatic filtering or matched white blood cell analysis is not performed.

**False-negative results:** Negative liquid biopsy results should be interpreted with caution because the absence of detectable ctDNA does not necessarily exclude the presence of a genomic alteration. False-negative findings may occur when tumors release very low amounts of ctDNA into the bloodstream or when the detected variant falls below the analytical limit of detection. Therefore, when plasma testing is negative but clinical suspicion remains high, reflex tissue molecular testing should be performed if sufficient tissue is available; alternatively, repeat liquid biopsy may be considered.

**Extended turnaround time:** One of the major advantages of liquid biopsy is its ability to provide molecular information more rapidly than tissue-based NGS, particularly when tumor tissue is unavailable, insufficient, or when tissue acquisition is delayed. This can facilitate earlier identification of actionable genomic alterations and timely initiation of targeted therapies in patients with advanced NSCLC. However, the turnaround time may still be prolonged in certain circumstances, particularly when multiple rounds of ctDNA extraction and library preparation are required to obtain sufficient material for sequencing. In addition, bioinformatic processing, including data filtering, alignment to the reference genome, variant calling, and quality control, can be time-consuming due to the complexity of sequencing data. Therefore, although liquid biopsy often offers a faster alternative to tissue-based molecular profiling, its turnaround time remains dependent on sample quality, assay complexity, laboratory workflow, and bioinformatic analysis.

**Variant interpretation and assigning genetic alterations to pathogenicity or benignity:** Interpretation of molecular findings remains an important challenge in liquid biopsy. Some detected variants may have uncertain clinical significance, whereas others represent passenger mutations that do not influence treatment decisions. Accurate interpretation therefore requires standardized reporting criteria, validated bioinformatic pipelines, and multidisciplinary molecular tumor boards to distinguish clinically actionable alterations from incidental findings and ensure appropriate therapeutic recommendations. Assigning genetic alterations to pathogenicity or benignity relies on open-source data that is continuously updated by researchers. However, a major challenge lies in the limited availability of functional experimental data that clearly establish the causal relationship between specific genetic or epigenetic alterations and disease. This scarcity of evidence makes it difficult to accurately classify alterations as pathogenic or benign. Moreover, substantial research efforts are still required in this field to improve our understanding and ensure more reliable interpretation.

**The cost of liquid biopsy:** The economic impact of liquid biopsy varies according to its clinical application. In patients with advanced NSCLC, plasma-based molecular profiling may be cost-effective by reducing the need for invasive tissue biopsies, shortening the time to treatment initiation, and enabling the early identification of actionable genomic alterations that guide targeted therapy and avoid other unnecessary costly treatments. In addition, the implementation of liquid biopsy as a population-level screening tool may offer substantially greater economic advantages. Although MCED assays and other screening-based liquid biopsy approaches require large-scale testing of asymptomatic individuals, resulting in considerable short-term costs for healthcare systems, they may substantially reduce long-term costs by decreasing healthcare expenditures. Notably, several strategies could be adopted to reduce testing costs, such as multiplex sequencing and sample pooling. Finally, comprehensive cost-effectiveness analyses incorporating diagnostic performance, downstream healthcare costs, reimbursement policies, and long-term clinical outcomes are still needed before liquid biopsy can be widely implemented for lung cancer screening.

**Economic and implementation challenges of liquid biopsy:** Although liquid biopsy has emerged as a promising tool for lung cancer diagnosis and management, its widespread implementation remains challenging, particularly in low- and middle-income countries. The high cost of next-generation sequencing, digital PCR platforms, specialized laboratory infrastructure, and bioinformatics support represents a major barrier to routine clinical adoption. In addition, limited access to advanced molecular diagnostics, scarcity of trained personnel, and the absence of standardized laboratory protocols further restrict the implementation of liquid biopsy in resource-limited settings. Reimbursement policies and regulatory frameworks also vary considerably between countries, affecting the accessibility and affordability of these technologies.

#### 3.3.5. Concordance and Discordance Between Liquid and Tissue Biopsy

When comparing genomic alterations identified by liquid biopsy and tissue biopsy, a high degree of concordance has been reported for several clinically actionable biomarkers, particularly in patients with advanced non-small cell lung cancer (NSCLC). Plasma circulating tumor DNA analysis has demonstrated high concordance with tissue-based molecular testing for alterations in genes including EGFR, ALK, ROS1, BRAF, MET, RET, and KRAS, supporting its clinical utility for molecular profiling and therapeutic decision-making when adequate tissue is insufficient or unavailable [33,34]. Several studies have investigated tissue concordance in representative lung cancer studies. For instance, Newman et al. reported a concordance of 91% using cancer personalized profiling by deep sequencing (CAPP-Seq) [58], whereas Zhang et al. investigated circulating tumor DNA (ctDNA) detection in patients with early-stage NSCLC and found that 31.2% (15/48) of matched tumor tissue–plasma pairs showed concordant mutations [59]. These results were obtained in different clinical and methodological contexts. Newman et al. evaluated CAPP-Seq across several NSCLC disease stages, including stage I–IV disease, and demonstrated ctDNA detection in 50% of stage I and 100% of stage II–IV patients, reflecting the influence of tumor burden and disease stage on ctDNA detectability [58]. In contrast, Zhang et al. specifically evaluated matched tumor tissue and preoperative plasma samples from patients with early-stage NSCLC using targeted next-generation sequencing and demonstrated that ctDNA detection and tissue–plasma concordance were influenced by histological subtype and tumor characteristics [59]. Therefore, the reported concordance values should not be interpreted as directly comparable measures of assay performance, but rather as estimates obtained under different disease-stage and methodological conditions. Liu et al. reported concordance values ranging from 85% to 98% using whole-genome bisulfite sequencing-based approaches [60]. Conversely, lower concordance rates have been described in studies involving early-stage disease or low circulating tumor DNA burden, such as those by Ooki et al. (31.3%) [5] and Jamal-Hanjani et al. (43%) [61], highlighting the influence of disease biology on assay performance. Nevertheless, concordance rates vary considerably among studies because they are influenced by several biological and technical parameters, including tumor stage, metastatic burden, tumor DNA shedding, assay sensitivity, sequencing methodology, and the time interval between tissue and blood sampling [33,34].

Discordant findings between tissue and liquid biopsy should not necessarily be interpreted as analytical inaccuracies. Instead, they frequently reflect the biological complexity of lung cancer, including both spatial and temporal tumor heterogeneity. A tissue biopsy sample detects only a limited region of a tumor at a single point in time, whereas ctDNA released into the bloodstream may originate from multiple primary and metastatic tumor sites, thereby offering a more comprehensive representation of the tumor’s genomic landscape. Consequently, liquid biopsy may detect emerging molecular alterations that are absent in the original tissue sample, particularly during disease progression or following therapy [62,63].

Despite the advantages of liquid biopsy, tissue biopsy remains the reference standard for histopathological diagnosis and assessment of tumor architecture. Current international clinical guidelines therefore recommend liquid biopsy as a complementary rather than an alternative diagnostic approach. In patients with advanced NSCLC, plasma-based molecular testing is particularly recommended when tissue is unavailable, insufficient, or when repeated tissue biopsy is not feasible. However, because low circulating tumor DNA levels may lead to false-negative plasma results, negative liquid biopsy findings should be confirmed by tissue testing whenever possible. The complementary use of tissue and liquid biopsy therefore maximizes the detection of clinically actionable genomic alterations and supports precision oncology in lung cancer management [34,64].

#### 3.3.6. Liquid Biopsy Test Improvement

Reliable detection of circulating tumor-derived biomarkers, particularly circulating tumor DNA (ctDNA), requires rigorous analytical validation to ensure that liquid biopsy assays perform with high accuracy and consistency under standardized laboratory conditions [24,65]. The principal analytical performance characteristics include analytical sensitivity, analytical specificity, accuracy, precision, repeatability, reproducibility, robustness, and limit of detection, all of which must be established before clinical implementation [64,65]. Highly sensitive molecular platforms such as digital droplet PCR (ddPCR), BEAMing, and next-generation sequencing (NGS) have enabled the identification of low-frequency somatic variants, with several validated NGS assays attaining limits of detection below 0.1% variant allele frequency while maintaining high analytical specificity [24,66]. However, assay performance is significantly influenced by pre-analytical variables such as methods and techniques used for blood collection, plasma separation time, cfDNA isolation procedures, sample storage conditions, sequencing depth, and bioinformatic analysis pipelines, highlighting the need for standardized workflows to ensure reproducible results across laboratories [64,65,67]. Moreover, biological factors including low ctDNA shedding in patients with early-stage lung cancer and the presence of clonal hematopoiesis of indeterminate potential may reduce analytical sensitivity or generate false-positive findings, thereby necessitating rigorous analytical validation and appropriate interpretation to ensure the reliability of the result [64,67,68,69,70].

Several improvements have been implemented to enhance the performance and reliability of liquid biopsy tests for cancer detection and genotyping. Experimental protocols were optimized with standardized procedures and specific buffers to ensure consistency, even when working with very low quantities of cell-free DNA. Typically, only a few nanograms of cfDNA are extracted from a relatively large blood volume (typically at least 20 mL). Given that tumor DNA released into the bloodstream often constitutes less than 5% of a patient’s total cfDNA, factors such as tumor type, stage (with stage IV yielding more cfDNA than early stages), and timing relative to surgery or treatment play a critical role in test yield and accuracy.

The quality and quantity of cfDNA should be carefully assessed using high-precision tools such as the Agilent Bioanalyzer and Qubit. Optimal cfDNA samples typically exhibit characteristic electropherogram peaks at approximately 145–180 bp, consistent with the size of tumor-derived DNA fragments. Rather than performing whole-genome sequencing (WGS), which interrogates the entire ~3.2-gigabase human genome, or whole-exome sequencing (WES), which focuses on the protein-coding regions (~1–2% of the genome), many cfDNA assays employ targeted sequencing panels. These panels analyze selected cancer-associated genes or genomic alterations relevant to early cancer detection. By restricting sequencing to clinically relevant regions, targeted approaches reduce costs while increasing sequencing depth, thereby improving the detection of low-frequency variants and subtle genomic alterations. Additionally, the integration of advanced molecular biology technologies further enhances assay sensitivity and specificity, representing a significant advance in cfDNA-based cancer detection.

Recent technological advances have substantially improved the analytical performance of liquid biopsy assays. The incorporation of unique molecular identifiers (UMIs) and advanced error suppression algorithms enables the discrimination of true low-frequency variants from sequencing artifacts, thereby increasing analytical sensitivity and specificity. Furthermore, duplex sequencing, which independently sequences both strands of a DNA molecule, further reduces sequencing errors and improves the detection of rare somatic mutations, particularly in early-stage disease and minimal residual disease. Beyond mutation analysis, emerging approaches based on DNA methylation profiling and fragmentomics, which analyze cfDNA methylation and fragmentation patterns and fragment size distributions, have demonstrated improved performance for early cancer detection and tissue-of-origin prediction. In addition, multi-analyte assays integrating genomic alterations with DNA methylation, circulating proteins, exosomal biomarkers, and circulating microRNAs are being developed to improve diagnostic accuracy compared with single-biomarker approaches. For MRD assessment, two smart approaches were designed to improve liquid biopsy, either in broad-spectrum detection or in specificity. The first is the tumor-informed assay, which is personalized using genomic alterations identified from the patient’s tumor tissue; this analysis makes the test more personalized. The second is the broader, tumor-naïve assay, which uses predefined biomarker panels without prior tumor sequencing, allowing detection of new tumor or metastasis driver variants. Both assays are under active investigation. Although these technological advances have significantly improved analytical performance, their clinical implementation still requires external validation in large prospective multicenter cohorts to demonstrate reproducibility, clinical validity, and clinical utility before widespread adoption in routine clinical practice [34,38,66].

Recent advances in artificial intelligence (AI), in particular machine learning (ML) and in deep learning (DL), have considerably expanded the potential of liquid biopsy both for lung cancer diagnosis and patient management. These methodologies effectively leverage the large volume and the complexity of the molecular genomic, epigenomic, transcriptomic, proteomic, and metabolomic data generated by liquid biopsy analyses. The result is the identification of complex disease-associated patterns by using the information on specific biomarker combinations, thereby facilitating differentiation of malignant from benign lesions, prediction of treatment response, and stratification of patients by risk of disease progression. Ultimately, integrating information from multi-comics provides a more comprehensive representation of complex systems like the tumor microenvironment.

The current literature indicates that applying AI-driven analyses to multi-omics data significantly improves diagnostic sensitivity and specificity, particularly for early-stage lung cancer, enhances prognostic assessment, and enables personalized therapeutic decision-making. Despite these promising advances, several challenges remain, including data standardization, model interpretability, external validation, and the integration into routine clinical workflows. These key obstacles include: (1) the size of available learning data, a key parameter in deciding whether to use DL methods, more powerful in their ability to find patterns, but also more data-greedy, or the more data-parsimonious ML methods; (2) the quality of the experimental data, which can strongly skew the AI-learning process; and (3) the ‘Black Box’ vs. Clinician Trust problem, undermining the regulatory bodies (e.g., the FDA) and oncologists’ trust in DL models, since it is usually difficult for the latter to understand why a patient has been assigned by the algorithm to a given risk class. Therefore, large prospective multicenter studies are needed for AI-assisted multi-omics liquid biopsy platforms to be routinely adopted in clinical practice [27,34,66].

Last but not least, AI algorithms offer the operational flexibility to optimize discovery strategies toward specific clinical endpoints, such as maximizing specificity for early-detection screening or prioritizing sensitivity during post-diagnostic therapeutic monitoring.

#### 3.3.7. Regulatory Considerations and Implementation of Liquid Biopsy into Routine Clinical Practice

The successful integration of liquid biopsy into routine clinical practice in lung cancer depends on a robust regulatory framework that ensures analytical validity, clinical validity, and clinical utility before an assay can be adopted for patient management [33,65]. Regulatory agencies and professional organizations, such as the U.S. Food and Drug Administration (FDA), the International Association for the Study of Lung Cancer (IASLC), the American Society of Clinical Oncology (ASCO), the College of American Pathologists (CAP), and the European Society for Medical Oncology (ESMO), have established recommendations to ensure that liquid biopsy assays meet rigorous standards for accuracy, reliability, reproducibility, and clinical performance [65]. These recommendations require manufacturers and clinical laboratories to validate assay performance, while also controlling pre-analytical variables, which can significantly influence test performance [33,65].

Regulatory approval of liquid biopsy assays also requires evidence that the detected genomic alterations provide clinically actionable information capable of improving therapeutic decision-making [2,34]. Consequently, several plasma-based diagnostic assays have received FDA approval for identifying actionable genomic alterations in patients with advanced non-small cell lung cancer (NSCLC), including EGFR mutations, allowing clinicians to select appropriate tyrosine kinase inhibitors [71]. Nevertheless, regulatory guidelines consistently emphasize that plasma testing should complement rather than completely replace tissue biopsy because of limited circulating tumor DNA shedding, particularly in patients with low tumor burden or early-stage disease [34,65].

Despite these advances, several regulatory and practical limitations continue to limit universal clinical implementation of liquid biopsy in lung cancer management. Considerable inter-laboratory variability exists because of several aspects, including differences in assay design, sequencing platforms, and bioinformatic pipelines [33,56]. Moreover, recent FDA oversight of laboratory-developed tests is intended to strengthen quality assurance, traceability, and clinical evidence requirements for liquid biopsy assays, thereby improving consistency and patient safety across healthcare systems [56]. As additional prospective clinical trials continue to demonstrate its clinical benefits, and with the evolution of new cutting-edge tools, regulatory agencies are expected to broaden the approved indications for liquid biopsy, facilitating its transition from a complementary diagnostic tool to an integral component of precision medicine and routine lung cancer management [33,34,56].

## 4. Conclusions

LDCT remains the reference standard for lung cancer screening, having demonstrated a significant reduction in lung cancer-specific mortality among high-risk individuals. However, its limitations, including false-positive findings, over-diagnosis, cumulative radiation exposure, and the inability to provide molecular information, highlight the need for complementary diagnostic approaches. Liquid biopsy has emerged as a promising minimally invasive tool that offers unique molecular insights and has already demonstrated clinical utility for molecular profiling and treatment selection in advanced NSCLC. In contrast, its applications in early detection, population screening, MRD assessment, and treatment monitoring remain under active investigation. Although advances in multi-analyte biomarkers, artificial intelligence, and multi-omics integration are expected to improve diagnostic performance, several unresolved challenges remain before liquid biopsy can be considered for wider adoption in lung cancer management. These include the very low tumor fraction in early-stage disease, which limits assay sensitivity and may result in false-negative findings; false-positive results associated with clonal hematopoiesis; and variability in assay performance, pre-analytical procedures, and interpretation, which may affect concordance across different platforms.

A phased research strategy is therefore required to establish the clinical role of liquid biopsy in different lung cancer settings. In the short term (approximately 1–3 years), research should prioritize analytical standardization, including harmonization of pre-analytical procedures, assay workflows, quality-control criteria, and reporting standards, while further improving assay sensitivity and specificity for low-tumor-fraction samples. In the intermediate term (up to 5 years), prospective, multicenter studies should evaluate the clinical performance of liquid biopsy in defined settings, particularly its integration with LDCT for lung cancer screening and its potential to reduce false-positive findings and unnecessary follow-up procedures. In parallel, prospective studies should further evaluate liquid biopsy for MRD detection and treatment monitoring using standardized clinical endpoints. In the longer term (beyond 10 years), adequately powered prospective trials should determine whether liquid-biopsy-guided strategies improve clinically relevant outcomes, including reductions in mortality and improved survival, and establish how these approaches can be integrated into routine lung cancer screening, surveillance, and precision oncology settings.

This staged approach would allow technical and analytical limitations to be addressed before large-scale clinical implementation and would provide the evidence needed to determine which liquid biopsy applications offer meaningful clinical benefit beyond current standards of care. Future large-scale multicenter prospective studies are needed to establish the clinical utility of liquid biopsy for lung cancer screening and to define its optimal integration with LDCT within precision oncology strategies.

## Figures and Tables

**Figure 1:**
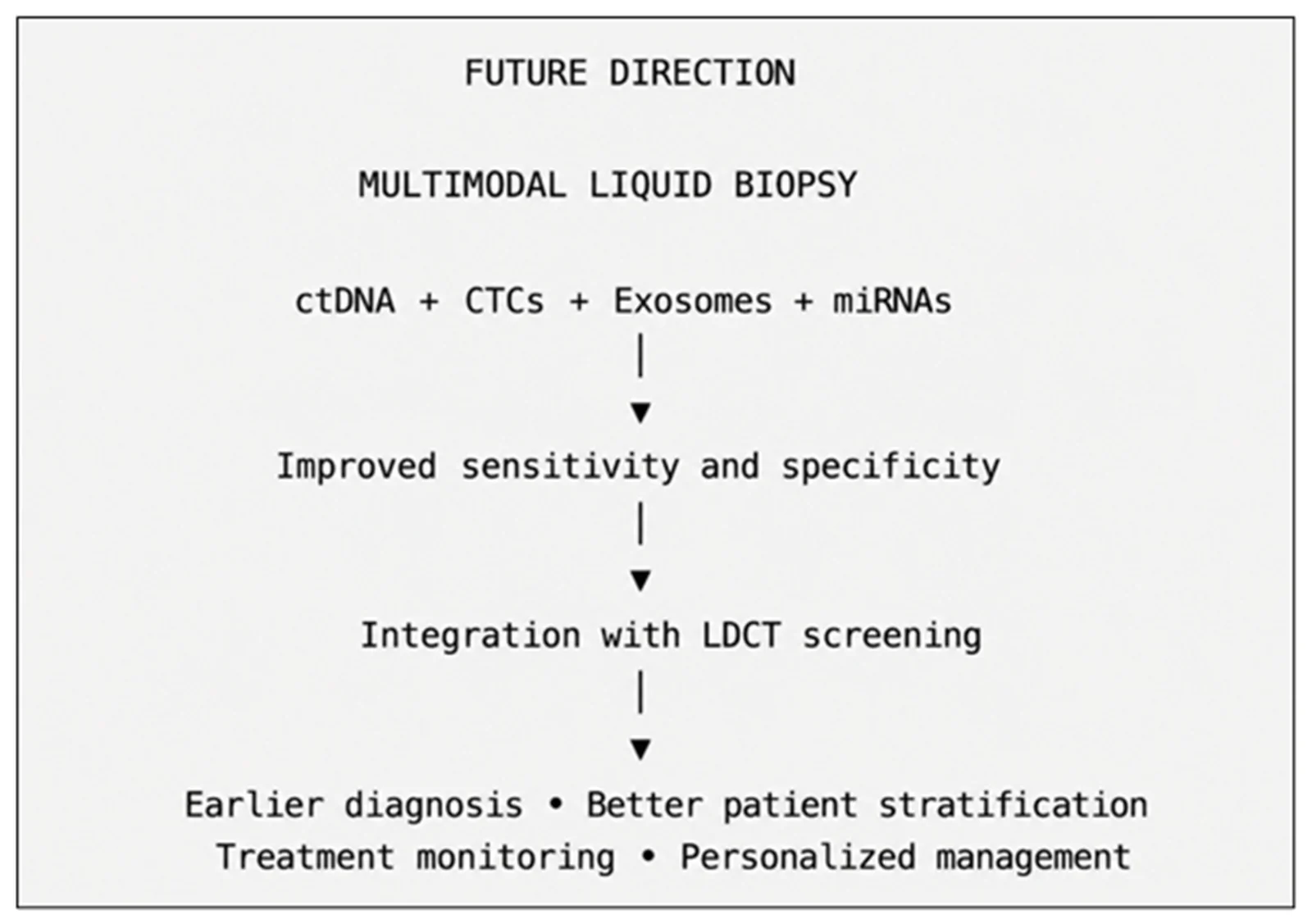
Future perspectives of multimodal liquid biopsy integration for lung cancer management.

**Figure 2:**
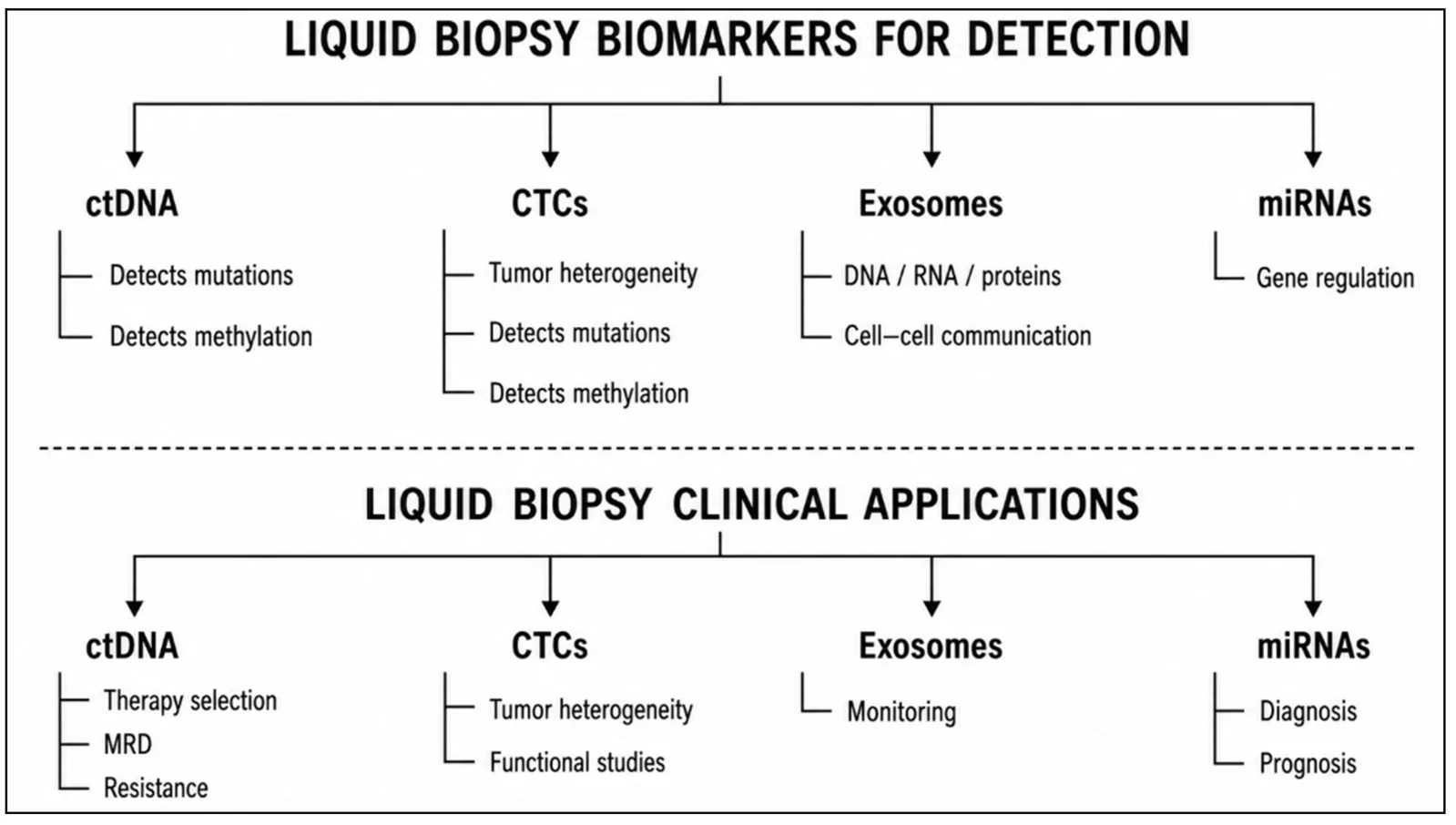
Major liquid biopsy biomarkers in lung cancer and their clinical utility.

**Figure 3:**
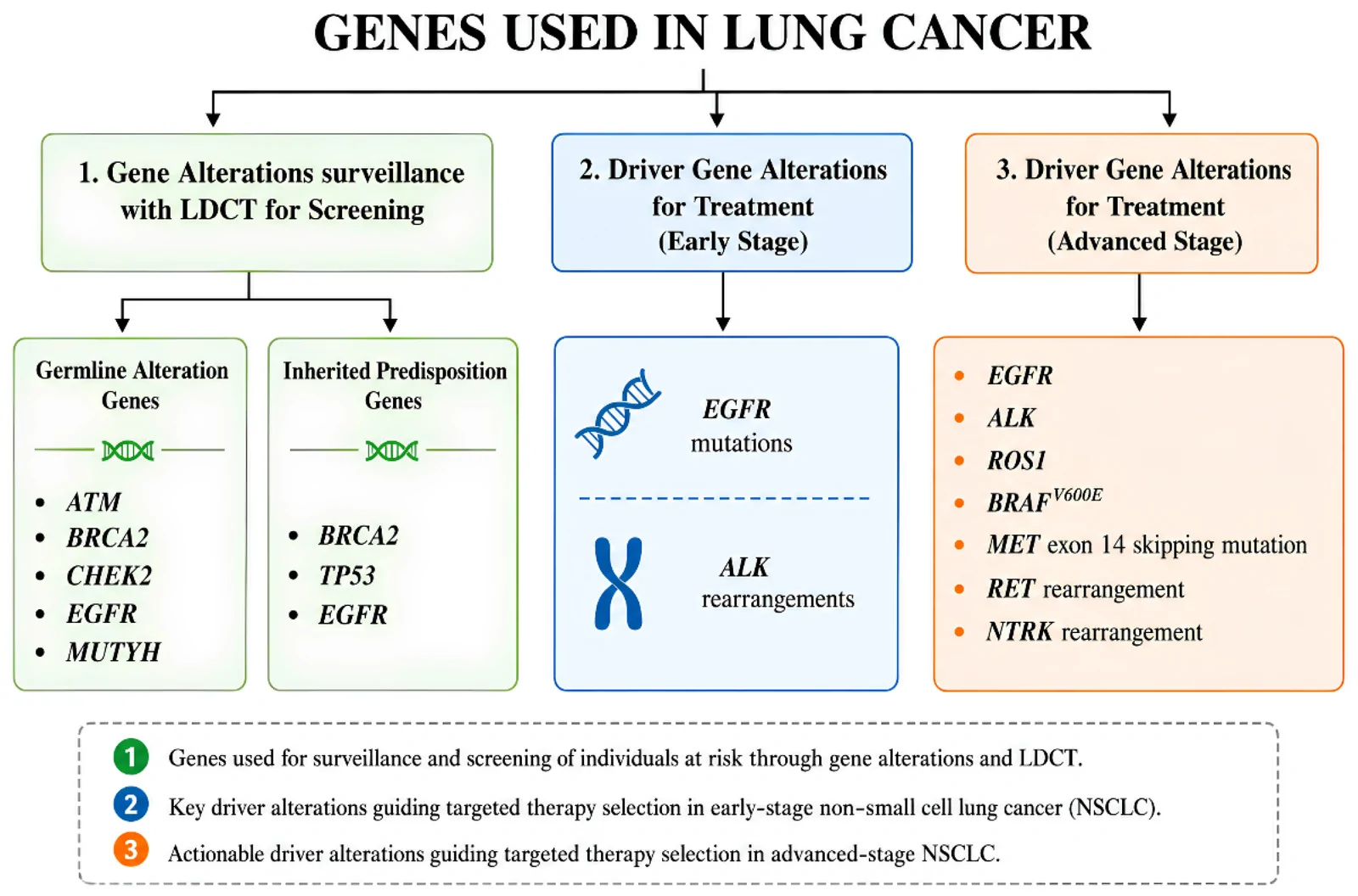
Clinical classification of molecular biomarkers used in lung cancer for diagnosis, prognosis, and therapeutic decision-making.

**Table 1:** Major randomized clinical trials evaluating LDCT for lung cancer screening.

Trial	Country	Population	Participant	Screening/Comparator Arm	Follow-Up Duration	Main Findings	Mortality Reduction	Major Limitations	Reference
NLST	USA	High-risk smokers (55–74 y, ≥30 pack-years)	53,454	LDCT vs. chest radiography, three annual screening rounds	Median 6.5 years	LDCT detected more early-stage cancers than chest radiography	20% LC mortality reduction	High false-positive rate	[9]
NELSON	The Netherlands & Belgium	High-risk smokers	15,789	Volume-based LDCT vs. no screening; screening at baseline and subsequent years using volumetric nodule assessment	~10 years	Volume-based nodule assessment reduced false positives	Significant LC mortality reduction	Implementation requires specialized protocols	[13]
MILD	Italy	High-risk smokers	NA	Annual or biennial LDCT vs. control/no screening	10 years	Long-term LDCT screening feasible	Trend toward mortality reduction	Limited statistical power	[14]
LUSI	Germany	High-risk smokers	4052	Annual LDCT vs. no screening, five annual screening rounds	Mean 8.8 years	Improved early detection	Trend toward mortality reduction	NA	[15]
Meta-analysis	Multiple countries	NA	84,558–90,475	NA	NA	Confirmed LDCT benefit	17–19% mortality reduction	Heterogeneity among studies	[16,17]

**Table 2:** Comparison of liquid biopsy biomarkers in lung cancer.

Feature	ctDNA	CTCs	Exosomes	miRNAs	DNA Methylation Markers
Sensitivity	Moderate in early-stage disease; high in advanced disease due to increased tumor DNA shedding	Generally low in early-stage LC because CTCs are extremely rare	Moderate to high; may outperform ctDNA in early-stage disease because exosomes are abundant	Moderate to high when using biomarker panels rather than single miRNAs	Moderate to high, particularly when multiple methylation markers are combined
Specificity	High for tumor-specific mutations	High when CTCs are accurately identified	Moderate; exosomes are released by both normal and tumor cells, requiring tumor-specific markers	Moderate; expression can be influenced by inflammation and other diseases	High because cancer-associated methylation patterns are relatively tumor-specific
Detection threshold	Low abundance in early-stage tumors requires highly sensitive techniques (ddPCR, NGS)	Very low (1–10 cells/7.5 mL blood); technically challenging	High abundance in plasma; easier to detect than CTCs	High abundance and remarkable stability in plasma	Low DNA input sufficient, but requires highly sensitive methylation assays
Cost	Moderate to high	High	Moderate to high	Relatively low to moderate	Moderate
Reproducibility	Good with standardized commercial assays	Limited because isolation methods vary considerably	Moderate; lack of standardized isolation protocols	Moderate; affected by pre-analytical and normalization methods	Good analytical reproducibility, but limited by lack of assay standardization
Main strengths	Detects actionable mutations; treatment selection; resistance monitoring; MRD detection	Provides intact viable cells for genomic, transcriptomic and phenotypic analyses	Protect nucleic acids from degradation; contain DNA, RNA and proteins; reflect tumor microenvironment	Highly stable; minimally invasive; potential for early detection	Excellent biological stability; promising for early cancer detection
Main limitations	Low ctDNA levels in early-stage disease; false negatives; interference from clonal hematopoiesis	Extremely rare cells; difficult isolation; epithelial-to-mesenchymal transition reduces capture efficiency	Isolation remains labor-intensive; co-isolation of contaminants; lack of standardization	Lack of standardized extraction, normalization and cut-off values	Marker heterogeneity; lack of universally validated methylation panels
Current clinical application	Routine clinical use for molecular profiling in advanced NSCLC and monitoring resistance mutations	Mainly prognostic and research applications	Research; potential diagnostic and prognostic biomarker	Research; promising for diagnosis and prognosis	Emerging application for early detection and screening
Regulatory approval	FDA-approved assays available (Cobas EGFR Mutation Test v2, Guardant360 CDx, FoundationOne Liquid CDx)	No FDA-approved assay for lung cancer	No FDA-approved assay for lung cancer	No FDA-approved assay for lung cancer	No FDA-approved methylation assay for lung cancer

**Table 3:** FDA-approved plasma-based companion diagnostic assays for NSCLC and their associated targeted therapies.

Diagnostic Assay (Manufacturer)	Indication	Sample Type	Associated Therapy Drug Trade Name (Generic)	Biomarker	FDA Approval Clearance	Grant Date
Agilent Resolution ctDx FIRST assay (Resolution Bioscience, Inc., San Diego, CA, USA)	NSCLC	Plasma	Krazati (adagrasib) NDA 216340	KRAS G12C	P210040	12 December 2022
cobas EGFR Mutation Test v2 (Roche Molecular Systems, Inc., Pleasanton, CA, USA)	NSCLC	Plasma	Tagrisso (osimertinib)	EGFR (HER1)—Exon 19 deletion or exon 21 L858R substitution mutation	P120019/S018	18 April 2018
cobas EGFR Mutation Test v2 (Roche Molecular Systems, Inc., Pleasanton, CA, USA)	NSCLC	Plasma	Tarceva (erlotinib)	EGFR (HER1)—Exon 19 deletion or exon 21 L858R substitution mutation	P150047	1 June 2016
cobas EGFR Mutation Test v2 (Roche Molecular Systems, Inc., Pleasanton, CA, USA)	NSCLC	Plasma	Tagrisso (osimertinib)	EGFR (HER1)—T790M	P150044	28 September 2016
FoundationOne Liquid CDx (Foundation Medicine, Inc., Cambridge, MA, USA)	NSCLC	Plasma	Iressa (gefitinib)	EGFR (HER1)—Exon 19 deletion or exon 21 L858R substitution mutation	P190032 P190032/S008	26 August 2020 19 December 2022
FoundationOne Liquid CDx (Foundation Medicine, Inc., Cambridge, MA, USA)	NSCLC	Plasma	Tagrisso (osimertinib)	EGFR (HER1)—Exon 19 deletion or exon 21 L858R substitution mutation	P190032 P190032/S008	26 August 2020 19 December 2022
FoundationOne Liquid CDx (Foundation Medicine, Inc., Cambridge, MA, USA)	NSCLC	Plasma	Tarceva (erlotinib)	EGFR (HER1)—Exon 19 deletion or exon 21 L858R substitution mutation	P190032 P190032/S008	26 August 2020 19 December 2022
FoundationOne Liquid CDx (Foundation Medicine, Inc., Cambridge, MA, USA)	NSCLC	Plasma	Tabrecta (capmatinib)	MET single nucleotide variants and indels that lead to MET exon 14 skipping	P190032/S001	15 July 2021
FoundationOne Liquid CDx (Foundation Medicine, Inc., Cambridge, MA, USA)	NSCLC	Plasma	Rozlytrek (entrectinib)	ROS1 fusions	P190032/S004	22 December 2022
FoundationOne Liquid CDx (Foundation Medicine, Inc., Cambridge, MA, USA)	NSCLC	Plasma	BRAFTOVI (encorafenib) in combination with MEKTOVI (binimetinib)	BRAF V600E	P190032/S011	11 October 2023
FoundationOne Liquid CDx (Foundation Medicine, Inc., Cambridge, MA, USA)	NSCLC	Plasma	Alecensa (alectinib)	ALK rearrangements	P200006	26 October 2020
FoundationOne Liquid CDx (Foundation Medicine, Inc., Cambridge, MA, USA)	NSCLC	Plasma	Tepmetko (tepotinib)	MET single nucleotide variants and indels that lead to MET exon 14 skipping	P190032/S015	14 November 2024
FoundationOne Liquid CDx (Foundation Medicine, Inc., Cambridge, MA, USA)	Solid tumors including NSCLC	Plasma	Rozlytrek (entrectinib)	NTRK1, NTRK2, and NTRK3 fusions	P190032/S004	22 December 2022
Guardant360 CDx (Guardant Health, Inc., Palo Alto, CA, USA)	NSCLC	Plasma	Tagrisso (osimertinib)	EGFR exon 19 deletions, EGFR exon 21 L858R, and T790M	P200010	7 August 2020
Guardant360 CDx (Guardant Health, Inc., Palo Alto, CA, USA)	NSCLC	Plasma	Rybrevant (amivantamab)	EGFR exon 20 insertions	P200010/S001	21 May 2021
Guardant360 CDx (Guardant Health, Inc., Palo Alto, CA, USA)	NSCLC	Plasma	Lumakras (sotorasib)	KRAS G12C	P200010/S002	28 May 2021
Guardant360 CDx (Guardant Health, Inc., Palo Alto, CA, USA)	NSCLC	Plasma	ENHERTU (fam-trastuzumab deruxtecan-nxki)	ERBB2 Activating Mutations (SNVs And Exon 20 Insertions)	P200010/S008	11 August 2022

## Data Availability

No new data were created or analyzed in this study. Data sharing is not applicable to this article.

## Supplementary Materials

The following supporting information can be downloaded at: https://www.mdpi.com/article/10.3390/cancers18193140/s1, Table S1: Summary of clinical trials assessing the clinical utility and validation of liquid biopsy approaches in lung cancer.

## Abbreviations

The following abbreviations are used in this manuscript:

AATS	American Association of Thoracic Surgery
ACS	American Cancer Society
ACCP	American College of Chest Physicians
CCO	Cancer Care Ontario
LDCT	low-dose computed tomography
USPSTF	United States Preventive Services Task Force
LC	Lung cancer
FDA	The Food and Drug Administration
TMB	Tumor Mutational Burden
MSI-H	High Microsatellite Instability
FFPE	Formalin-Fixed Paraffin-Embedded
ASCO	American Society of Clinical Oncology
ESMO	European Society for Medical Oncology
MCED	Multi-Cancer Early Detection
MS	Mass Spectrometry
MRD	Minimal Residual Disease
NGS	Next-Generation Sequencing
NSCLC	Non-Small Cell Lung Cancer
ctDNA	Circulating Tumor DNA
CE-IVD	Conformité Européenne In Vitro Diagnostic
Pet/Ct	Positron Emission Tomography Computed Tomography
SCLC	Small Cell Lung Cancer
