# Supplementary material for: Refining Lung Cancer Care: The Emerging Role of Liquid Biopsy in Screening, Diagnosis, and Precision Oncology
Source: Cancers (Basel). 2026 Sep 28;18(19):3140. doi: 10.3390/cancers18193140 (PMC13663976; doi:10.3390/cancers18193140)
Supplement: Supplementary file 1 [file cancers-18-03140-s001.zip › cancers-4466793-supplementary.pdf]

cancers

MDPI

Supplementary Material

Table S1. Summary of clinical trials assessing the clinical utility and validation of liquid biopsy approaches in lung cancer.

Clinical Trial ID	Study Title	Study design	Phase	Number of study locations	Tumor Subtype	Disease stage	Number of patients	Liquid biopsy analyte	Test Type	Clinical purpose	Approach used	Genes tested	Tissue concordance	Results overview	Starting date	Research completion or estimation
NCT02511288	Liquid Biopsies in Patients Presenting Non-small Cell Lung Cancer (LIBIL)	Observational	NA	8	NSCLC	Stage IIIb Stage IV	900	ctDNA	Genetic	Detection	DdPCR targeted NGF, whole exome sequencing	EGFR, KRAS, BRAF, HER2, ALK, ROS1 and MET alterations	NA	No study results posted on ClinicalTrials.gov for this study (Recruiting)	Jul-2015	Dec-2026
NCT04258137	Circulating DNA to Improve Outcome of Oncology PatiEnt. A Randomized Study (COPE)	Interventional	Phase II	6	NSCLC	NA	332	ctDNA	Genetic	Follow-up	NA	NA	NA	No study results posted on ClinicalTrials.gov for this study (Recruiting)	Sep - 2020	Dec-2027
NCT03178552	A Study to Evaluate the Efficacy and Safety of Multiple Targeted Therapies as Treatments for Participants With Non-Small Cell Lung Cancer (NSCLC) (B-FAST)	Interventional	Phase II Phase III	164	NSCLC	Stage IIIb Stage IV	1000	cfDNA	Genetic	Treatment	NGS	NA	NA	No study results posted on ClinicalTrials.gov for this study (No longer looking for participants)	Sep-2017	Oct-2026
NCT04790682	Liquid Biopsy to prEdict Responses To First-line immunotherapY in Metastatic Non-small Cell LUNG Cancer. LIBERTY LUNG (LIBERTYLUNG)	Interventional	NA	3	NSCLC	metastatic stage	300	ctDNA	Genetic	Follow-up	NGS	NA	NA	No study results posted on ClinicalTrials.gov for this study	May-2021	Jun-2030
NCT04698681	NGS Screening Protocol to Detect Mutation of KEAP1 or NRF2/ NFE2L2 Genes for the KEAPSAKE (CX-839-014) Trial	Observational	NA	63	NSCLC	Stage IV	20	ctDNA	Genetic	Screening	NGS	KEAP1 or NRF2/NFE2L2	NA	No study results posted on ClinicalTrials.gov for this study (Lack of Clinical Benefit - No longer looking for participants)	Jan-2021	Feb-2022
NCT03721120	Evaluation of the Feasibility and Clinical Relevance of Liquid Biopsy in Patients With Suspicious Metastatic Lung Cancer (LIBELULE)	Randomized	Phase III	16	NSCLC	metastatic stage	319	cfDNA	Genetic	Treatment	InVision	panel of 35 genes	NA	No study results posted on ClinicalTrials.gov for this study (No longer looking for participants)	Apr-2019	Jul-2023

NCT04703153	LIQUIK: LIQUId Biopsy for Detection of Actionable Genomic BiomarKers in Patients With Advanced Non-small Cell Lung Cancer (NSCLC) (LIQUIK)	Observational	NA	5	NSCLC	metastatic stage	151	cfDNA	Genetic	Detection	NGS	EGFR, ALK, RET, ROS1, NTRK fusions, MET, BRAF, ERBB2 and Kras	72.1%	No study results posted on ClinicalTrials.gov for this study	Apr-2021	Dec-2024
NCT04863924	Accelerating Lung Cancer Diagnosis Through Liquid Biopsy (ACCELERAT	Observational	NA	1	NSCLC	Stage III Stage IV	170	ctDNA	NA	Diagnosis	NA	NA	NA	No study results posted on ClinicalTrials.gov for this study	Jun-2021	Dec-2026
NCT03865511	MEchanisms of Resistance in EGFR Mutated Nonpretreated Advanced Lung Cancer Receiving OSimErtib (MELROSE)	Interventional	Phase II	17	NSCLC	Stage IIIb Stage IV	150	ctDNA	Genetic	Treatment	dPCR, NGS	EGFR	NA	No study results posted on ClinicalTrials.gov for this study	Apr-2019	Jun-2025
NCT02864992	Tepotinib Phase II in NSCLC Harboring MET Alterations (VISION)	Interventional	Phase II	176	NSCLC	Stage IIIb Stage IV	337	cfDNA	Genetic	Treatment	NA	METex14 Skipping Alterations or MET Amplification	89%	Tepotinib showed significant activity in MET exon 14-altered NSCLC, with similar response rates identified by liquid biopsy and tissue biopsy (48% vs 50%), supporting cfDNA testing for molecular profiling and treatment selection	Sep-2016	Apr-2026
NCT04265534	KEAPSAKE: A Study of Telaglenastat (CB-839) With Standard-of-Care Chemoimmunotherapy in 1L KEAP1/ NRF2-Mutated, Nonsquamous NSCLC (KEAPSAKE)	Randomized	Phase II	102	NSCLC	NA	40	ctDNA	Genetic	Treatment	NGS	KEAP1/ NRF2	NA	No study results posted on ClinicalTrials.gov for this study (Lack of Clinical Benefit - No longer looking for participants)	Jul-2020	Feb-2022
NCT03940703	A Study of Tepotinib Plus Osimertinib in Osimertinib Relapsed MET Amplified NSCLC (INSIGHT 2)	Interventional	Phase II	179	NSCLC	metastatic stage	140	ctDNA	Genetic	Treatment	NGS	EGFR, MET Amplification	NA	Tepotinib plus osimertinib achieved a 50% objective response rate in patients with MET-amplified NSCLC after osimertinib progression, supporting MET amplification as a therapeutic target	Sep-2019	May-2026

https://doi.org/10.3390/cancers18193140

NCT03644823	Combinatory ImmunoTherapy-1 (Com-IT-1) Irradiation and PD-1 Blockade in Locally Advanced / Advanced NSCLC (COM-IT-1)	Interventional	Phase II	1	NSCLC	Stage III Stage IV	21	ctDNA	Genetic	Treatment	NA	NA	NA	ComIT-1 demonstrated the feasibility of atezolizumab plus SBRT in advanced NSCLC, with ctDNA reduction showing potential as an early biomarker of treatment response during immunotherapy.	Aug-2018	Dec-2020
NCT03740126	Surveillance With PET/ CT and Liquid Biopsies of Stage I-III Lung Cancer Patients After Completion of Definitive Therapy (SUPE_R)	Interventional	NA	3	NSCLC	Stage I-III	750	ctDNA	NA	Follow-up	NA	NA	NA	No study results posted on ClinicalTrials.gov for this study	Oct-2018	Aug-2026
NCT04093167	Study of CTDNA Response Adaptive Immuno-Chemotherapy in Lung Cancer	Interventional	Phase II Phase III	11	Lung cancer	Stage III	230	ctDNA	NA	Treatment	NA	NA	NA	No study results posted on ClinicalTrials.gov for this study (Recruiting)	May-2020	Jul-2027
NCT04966663	Using ctDNA to Determine Therapies for Lung Cancer (ctDNA Lung RCT)	Interventional	Phase II	1	NSCLC	early stage	66	ctDNA	NA	Treatment	NA	NA	NA	No study results posted on ClinicalTrials.gov for this study (Recruiting)	Mar-2022	Dec-2026
NCT05254782	Using ctDNA to Detect Minimal Residual Disease After Lung Cancer Resection	Observational	NA	3	NSCLC	Early stage (T1-T4 (T3,T4 multifocal) N0M0)	360	ctDNA	NA	Detection MRD	NA	NA	NA	No study results posted on ClinicalTrials.gov for this study (Recruiting)	Jul-2021	Mar-2028
NCT04712877	Testing Tumor Tissue and Blood to Help Select Personalized Treatments for Patients With Suspected Lung Cancers	Observational	NA	21	NSCLC	Early stage	1000	ctDNA	Genetic	Screening	NGS	EGFR, BRAF, MET, KRAS, HER2, ALK, RET, NTRK, ROS, amplification of MET and HER2	NA	No study results posted on ClinicalTrials.gov for this study (Recruiting)	Jun-2022	Jun-2026
NCT04162678	Fluid Biopsy for the Diagnosis of Lung Cancer	Observational	NA	2	Lung cancer	NA	51	CtDNA CTC	NA	Diagnosis	NA	NA	NA	No study results posted on ClinicalTrials.gov for this study (insufficient accrual - No longer looking for participants)	Jan-2020	Dec-2024
NCT05384769	Feasibility of Cell-Free DNA Liquid Biopsy in Screening High-Risk Patients for Lung Cancer	Interventional	NA	1	Lung cancer	NA	108	CfDNA	NA	Screening	NA	NA	NA	No study results posted on ClinicalTrials.gov for this study (Recruiting)	Jul-2022	Jan-2029

NCT07432308	Liquid Biopsy Genotyping Using Oncomine Precision Assay GX in Advanced NSCLC (Liquid biopsy)	Interventional	NA	1	NSCLC	Advanced stage	22	CtDNA	Genetic	Diagnosis	Oncomine Precision Assay GX method	NA	NA	No study results posted on ClinicalTrials.gov for this study (No longer looking for participants)	Feb-2024	Aug-2025
NCT06707519	NSCLC Liquid Biopsies and Exhaled VOC	Observational	NA	1	NSCLC	NA	210	CtDNA	Genetic	Follow-up	NA	NA	NA	No study results posted on ClinicalTrials.gov for this study (Recruiting)	Feb-2023	Dec-2027
NCT06081270	Prospective Study on Resistance-associated Mutations in Metastatic Lung Cancer (MUT-ONC)	Observational	NA	1	NSCLC	NA	20	CtDNA	Genetic	Treatment	NGS	EGFR	NA	No study results posted on ClinicalTrials.gov for this study (Unknown status)	Dec-2021	Dec-2024
NCT03479099	Liquid Biopsy in Lung Cancer	Observational	NA	1	Lung cancer	NA	111	CtDNA<br>CTC	NA	Diagnosis	NA	NA	NA	No study results posted on ClinicalTrials.gov for this study (No longer looking for participants)	Mar-2018	Mar-2019
NCT04474613	Liquid Biopsy to Reduce Time to Treatment for Advanced Nonsquamous NSCLC Diagnosed at Outside Sites	Interventional	NA	1	NSCLC	Advanced stage	26	CtDNA	NA	Treatment	NA	NA	NA	No study results posted on ClinicalTrials.gov for this study (No longer looking for participants)	Aug-2020	Feb-2022
NCT07358715	Liquid Biopsy-Based Pre-Screening to Streamline LDCT Lung Cancer Screening in High-Risk Individuals	Interventional	NA	1	Lung cancer	Stage I-II<br>Stage III-IV	140	Exosome	NA	Screening	Exosome-Based Liquid Biopsy Assay	NA	NA	No study results posted on ClinicalTrials.gov for this study (Recruiting)	Nov-2024	Dec-2028
NCT05255133	A Study to Evaluate the Value of Circulating Tumour DNA in Follow-up of Patients With an Advanced Gastroenteropancreatic or Lung Neuroendocrine Tumour Under Everolimus +-SSA Treatment (Liquid-NET)	Interventional	NA	8	Lung Neuroendocrine Tumor	Advanced stage	22	CtDNA	Genetic<br>Epigenetic	Follow-up	NGS, dPCR, photoelectrochemical biosensor	NA	NA	No study results posted on ClinicalTrials.gov for this study (No longer looking for participants)	Sep-2019	Jun-2022
NCT05708599	A Study to Compare Tissue and Liquid Biopsies in People With Different Types of Cancer	Interventional	NA	1	NSCLC	NA	102	CtDNA	Genetic	Treatment	NA	NA	NA	No study results posted on ClinicalTrials.gov for this study (No longer looking for participants)	Mar-2023	Feb-2027
NCT05462795	Liquid Biopsy for Early Non-small Lung Cancer Detection	Observational	NA	1	NSCLC	NA	171	NA	Epigenetic	Detection	NA	NA	NA	No study results posted on ClinicalTrials.gov for this study (No longer looking	Aug-2022	Mar-2024

https://doi.org/10.3390/cancers18193140

														for participants)		
NCT05853887	Liquid Biopsy Based NGS in Newly Diagnosed NSCLC (iNUDGE)	Interventional	NA	6	NSCLC	NA	260	NA	NA	Diagnosis	NGS	NA	NA	No study results posted on ClinicalTrials.gov for this study (No longer looking for participants)	Jun-2023	DEC-2026
NCT04817046	Assessment of Early-detection Based on Liquid Biopsy in Lung Cancer (ASCEND-LUNG)	Observational	NA	1	Lung cancer	Early stage	467	CfDNA	Genetic	Detection	NA	NA	NA	No study results posted on ClinicalTrials.gov for this study (Unknown status	Feb-2021	Dec-2024
NCT07009769	AI-Driven Precision Diagnostic System for Lung Cancer Based on Liquid Biopsy	Observational	NA	1	Lung cancer	NA	865	Exosome	Genetic	Diagnosis	NA	NA	NA	No study results posted on ClinicalTrials.gov for this study (No longer looking for participants)	Oct-2024	May-2025
NCT04253509	Liquid Biopsy Using Methylation Sequencing for Lung Cancer	Observational	NA	1	Lung cancer	NA	280	CtDNA	Epigenetic	Diagnosis	NGS	NA	NA	No study results posted on ClinicalTrials.gov for this study (No longer looking for participants)	Feb-2020	Jan-2022
NCT06997458	Liquid BiOpsy, Lung and Colon Cancer in AndaluciA (LOLA-PROJECT)	Observational	NA	1	NSCLC	Stage IV	500	CtDNA	Genetic	Cancer management	NGS	NA	NA	No study results posted on ClinicalTrials.gov for this study (Enrolling by invitation)	Aug-2024	Jul-2026
NCT05254132	Artificial Intelligence for Help Non-Small Cell Lung Cancer: Measure Cancer Biology and Treatment Response Via Imaging (SALMON)	Observational	NA	NA	NSCLC	metastatic stage	1000	NA	Genetic	Treatment	NA	EGFR, ROS1, BRAF	NA	No study results posted on ClinicalTrials.gov for this study (Unknown status)	Jul-2022	Jun-2025
NCT05066776	Liquid Biopsy With PET/ CT Versus PET/ CT Alone in Diagnosis of Small Lung Nodules	Observational	NA	1	NSCLC	NA	31	CtDNA	NA	Diagnosis	NA	NA	NA	No study results posted on ClinicalTrials.gov for this study (No longer looking for participants)	Sep-2021	Feb-2023
NCT04566614	Preventing Viral Pandemic Associated Risk of Cancer Death Using Less Invasive Diagnostic Tests- Liquid Biopsies (PREVAILctDNA)	Observational	NA	1	Neoplasm of lung	Stage III Stage IV	128	CtDNA	Genetic	Diagnosis	Whole genome sequencing	NA	NA	No study results posted on ClinicalTrials.gov for this study (No longer looking for participants)	Jun-2020	Jan-2025

https://doi.org/10.3390/cancers18193140

NCT06889467	Liquid Biopsy Assay of EBUS-TBNA Supernatant Fluid for Diagnosing Lung Cancer	Observational	NA	1	NSCLC	NA	10	CfDNA	NA	Diagnosis	NGS	NA	NA	No study results posted on ClinicalTrials.gov for this study (Recruiting)	Jan-2025	Dec-2025
NCT05648370	Liquid Biopsy in Advanced Oligometastatic NSCLC Receiving Surgery	Observational	NA	1	NSCLC	Stage IV	60	CtDNA	Genetic	NA	Sequencing	NA	NA	No study results posted on ClinicalTrials.gov for this study (Unknown status)	Jul-2022	Jun-2025
NCT05432128	To Establish a Molecular Typing System for Early Diagnosis of Lung Cancer	Observational	NA	1	Lung cancer	NA	600	CtDNA	Epigenetic	Diagnosis	NA	NA	NA	No study results posted on ClinicalTrials.gov for this study (Unknown status)	Jan-2020	Dec-2025
NCT05935384	SIBYL: obServation of Therapy Response With lIquid BiopsY evaLuation	Observational	NA	1	NSCLC	Stage III Stage IV	470	CtDNA	Genetic	Treatment	Guardant 360	NA	NA	No study results posted on ClinicalTrials.gov for this study (Recruiting)	Oct-2023	Dec-2030
NCT07182214	An Observational Study on the Assessment of Lung Cancer Using Liquid Biopsy for Collecting miRNA Expression Profile and DNA Methylation Biomarkers.	Observational	NA	1	Lung cancer	NA	1340	CtDNA	Epigenetic	NA	NA	NA	NA	No study results posted on ClinicalTrials.gov for this study (Recruiting)	May-2024	Dec-2026
NCT06422637	A Prospective Interventional Study on Early Screening for Lung Cancer Using Liquid Biopsy	Interventional	NA	1	Lung cancer	Early stage	5944	CfDNA	Genetic	Screening	Whole genome sequencing	NA	NA	No study results posted on ClinicalTrials.gov for this study (Not yet Recruiting)	Jun-2024	Dec-2027
NCT03702309	Liquid Biopsy Evaluation and Repository Development at Princess Margaret (LIBERATE)	Interventional	NA	1	Lung cancer	NA	2500	CfDNA CfRNA	Genetic	Cancer management	NA	NA	NA	No study results posted on ClinicalTrials.gov for this study (Recruiting)	Aug-2017	Jul-2026
NCT05268952	A Study to Evaluate the Value of Circulating Tumour DNA in Follow-up of Patients With an Advanced Gastroenteropancreatic or Lung Neuroendocrine Tumour Under Everolimus +-SSA Treatment (Liquid-NET 2.0)	Interventional	NA	3	lung neuroendocrine tumors	Advanced stage	100	CtDNA	Genetic	Treatment	NA	NA	NA	No study results posted on ClinicalTrials.gov for this study (Unknown status)	May-2019	Dec-2025
NCT06105177	Implementation of Liquid Biopsy-based Decision Support Models - LM² Study (LM2)	Observational	NA	5	Lung cancer	Stage IIIb Stage IV	800	CtDNA CTC	Genetic	Cancer management	NGS	PD-L1	NA	No study results posted on ClinicalTrials.gov for this study (Recruiting)	Oct-2023	Oct-2026

NCT04566432	Therapeutic ResistAnce and Clonal Evolution Assessed With Liquid Biopsy in ICIs Treated NSCLC Patients	Observational	NA	1	NSCLC	Stage IIIb Stage IV	252	CtDNA	Genetic	Treatment	NA	ALK, ROS1, MET	NA	No study results posted on ClinicalTrials.gov for this study (No longer looking for participants)	Jul-2020	Dec-2025
NCT06228508	.Molecular Diagnosis of Lung Cancer Via Bronchoscopy: The Significance of Bronchial Liquid Biopsy (ctDNA and ctRNA) (BiliBro)	Observational	NA	1	NSCLC	NA	50	CtDNA CtRNA	Genetic	Treatment	NGS	NA	NA	No study results posted on ClinicalTrials.gov for this study (Unknown status)	Feb-2024	Aug-2024
NCT07475637	Liquid Biopsy Monitoring Of EGFR T790M in Advanced Non-Small Cell Lung Cancer (LBM-T790M)	Observational	NA	1	NSCLC	Stage IIIb Stage IV	70	CtDNA	Genetic	Detection monitoring	NA	EGFR		No study results posted on ClinicalTrials.gov for this study (No longer looking for participants)	Mar-2023	Dec-2025
NCT07649629	Impactof Liquid Biopsy on the Therapeutic Pathway for Lung Cancer: Advancing the Molecular Characterization of Patients With Advanced Lung Adenocarcinoma by Integrating Liquid Biopsy Into the Early Stages of the Diagnostic and Therapeutic Pathway (INNOVA-LUNG)	Observational	NA	1	NSCLC	Stage III Stage IV	70	NA	Genetic	Diagnosis Treatment	NGS	NA	NA	No study results posted on ClinicalTrials.gov for this study (Recruiting)	Sep-2025	Mar-2027
NCT05846594	A Study to Evaluate the Impact of Liquid Biopsy in Participants With a Clinical Diagnosis of Advanced Cancer (L1st)	Interventional	NA	18	Lung cancer	Advanced stage	463	NA	NA	Diagnosis	FoundationOne ® Liquid CDx	NA	NA	No study results posted on ClinicalTrials.gov for this study (No longer looking for participants)	Sep-2023	Sep-2024
NCT06849518	Study of Longitudinal Plasma PD-L1 Testing With Immunotherapy in Lung Cancer	Interventional	NA	1	NSCLC	Stage IIIb Stage IV	30	CtDNA CtRNA	Genetic	Treatment	NGS	PD-L1		No study results posted on ClinicalTrials.gov for this study (Recruiting)	Feb-2025	Mar-2027
NCT03971175	Influence of Biopsy Technique on Moleculargenetic Tumor Characterisation in NSCLC (PROFILER)	Interventional	NA	1	NSCLC	NA	540	NA	Genetic	NA	NA	NA	NA	No study results posted on ClinicalTrials.gov for this study (Unknown status)	Dec-2028	Jun-2023

https://doi.org/10.3390/cancers18193140

NCT05469022	Neoadjuvant Lazertinib Therapy in EGFR-Mutation Positive Lung Adenocarcinoma Detected by BALF Liquid Biopsy	Interventional	Phase II	1	NSCLC	Early stage	40	Exosome	Genetic	Treatment	NA	EGFR	NA	No study results posted on ClinicalTrials.gov for this study (Recruiting)	May-2022	May-2027
NCT05299125	Amivantamab, Lazertinib, and Pemetrexed for First-line Treatment of Recurrent/ Metastatic Non-small Cell Lung Cancers With Epidermal Growth Factor Receptor Mutations (AMIGO-1)	Interventional	Phase II	14	NSCLC	Early stage	54	NA	Genetic	Treatment	NA	EGFR	NA	No study results posted on ClinicalTrials.gov for this study (No longer looking for participants)	May-2023	Jul-2027
NCT04223596	Clinical Utility of Liquid Biopsy in Brigatinib ALK+ Patients (CUBIK)	Interventional	Phase II	15	NSCLC	Stage IIIb Stage IV	33	NA	Genetic	Treatment	NA	EML4-ALK	NA	No study results posted on ClinicalTrials.gov for this study (No longer looking for participants)	May-2020	Dec-2026
NCT04014465	Monitoring Efficacy of Radiotherapy in Lung Cancer and Esophageal Cancer	Observational	NA	1	Lung cancer	NA	150	CtDNA	NA	Treatment	NGS	NA	NA	No study results posted on ClinicalTrials.gov for this study (Unknown status)	Feb-2019	Dec-2022
NCT03397355	Monitoring the Changes of Tumor-related Biomarkers Before and After Pulmonary Nodule Biopsy.	Observational	NA	1	Lung cancer	Early stage	1000	CtDNA CTC	NA	Cancer management	NA	NA	NA	No study results posted on ClinicalTrials.gov for this study (Unknown status)	Jan-2018	Dec-2022
NCT07331701	Association of BAL Fluid T-Cell Immune Activity With Tumor PD-L1 Expression and Its Prognostic Value: A Prospective Observational Study	Observational	NA	1	Lung cancer	Stage IV	60	NA	NA	Treatment	NA	PD-L1	NA	No study results posted on ClinicalTrials.gov for this study (Not yet Recruiting)	Jan-2026	Dec-2027
NCT04624373	Genotyping of Ebus-tbna Supernant Cell-free Dna in Nsclc (CELTICS)	Observational	NA	1	NSCLC	Stage IV	50	CfDNA	Genetic	Diagnosis	NA	EGFR, HER2, BRAF, ALK, ROS1	NA	No study results posted on ClinicalTrials.gov for this study (Not yet Recruiting)	Apr-2021	Dec-2025
NCT04704960	CirculAting Tumor DNA in Patients DIagnosed With Lung Metastasis From Colorectal Cancer: candiDate Selection for Local AblaTive thErapy (CANDIDATE)	Observational	NA	1	Lung cancer	Metastatic stage	400	CfDNA	NA	Treatment	NGS	NA	NA	No study results posted on ClinicalTrials.gov for this study (Unknown status)	Feb-2021	Dec-2023

https://doi.org/10.3390/cancers18193140

NCT05889247	Circulating Tumor DNA Guided Treatment Monitoring in Advanced Lung Cancer (PRELUCA)	Interventional	NA	5	NSCLC	Advanced stage	350	CtDNA	NA	Treatment	NA	NA	NA	No study results posted on ClinicalTrials.gov for this study (Not yet Recruiting)	Jul-2023	Jun-2032
NCT04887545	Immune- and Microenvironment- Proteogenomics Profiling for Classifying Lung Cancer Patients	Observational	NA	1	Lung cancer	NA	200	NA	NA	Diagnosis	NA	NA	NA	No study results posted on ClinicalTrials.gov for this study (Unknown status)	Oct-2018	Dec-2022
NCT04912687	Implementing Circulating Tumor DNA Analysis at Initial Diagnosis to Improve Management of Advanced NSCLC Patients (CIRCULAR)	Interventional	NA	9	NSCLC	Advanced stage	581	CfDNA	Genetic	Cancer management	Sequencing	EGFR	NA	No study results posted on ClinicalTrials.gov for this study (No longer looking for participants)	Jan-2022	Jan-2026
NCT06573073	Evaluation of Clinical Performance and IVD Test of LifeOS Digital PCR Liquid Biopsy Kits for EGFR T790M Mutation	Observational	NA	1	NSCLC	Stage IIIb Stage IV	300	NA	Genetic	Detection	PCR	EGFR	NA	No study results posted on ClinicalTrials.gov for this study (Recruiting)	Sep-2024	Dec-2028
NCT06930807	Liquid Biopsy-Based Novel Modality for Postoperative Management of Lung Cancer	Interventional	NA	1	NSCLC	Stage-IIIb Stage IV	1000	NA	NA	Detection	NA	NA	NA	No study results posted on ClinicalTrials.gov for this study (Recruiting)	Mar-2025	Jul-2028
NCT05059444	ORACLE: Observation of ResiduAl Cancer With Liquid Biopsy Evaluation (ORACLE)	Observational	NA	57	NSCLC	Stage Ib-Stage III	2020	CtDNA	NA	Detection	Guardant	NA	NA	No study results posted on ClinicalTrials.gov for this study (Recruiting)	Sep-2021	Aug-2029
NCT06287593	The Feasibility of Dynamic Liquid Biopsy Monitoring During Neoadjuvant Treatment for EGFR-mutated NSCLC	Interventional	NA	NA	NSCLC	Stage II-IIIB	300	CtDNA	NA	Treatment	NA	EGFR	NA	No study results posted on ClinicalTrials.gov for this study (Not yet Recruiting)	Apr-2024	Dec-2027
NCT05560776	Optimizing Endobronchial Ultrasound Sampling for Molecular Markers for NSCLC (OPTIMAL)	Interventional	NA	1	NSCLC	NA	120	NA	NA	Cancer management	NGS	NA	NA	No study results posted on ClinicalTrials.gov for this study (No longer looking for participants)	May-2023	May-2026
NCT06869239	Prediction of Response to PD-L1 Inhibitor After Chemoradiotherapy in Limited-Stage Small-Cell Lung Cancer Using Multi-omics-based Liquid Biopsy	Observational	NA	1	SCLC	NA	65	NA	NA	Treatment	NA	PD-L1	NA	No study results posted on ClinicalTrials.gov for this study (Recruiting)	Feb-2025	Jan-2028

https://doi.org/10.3390/cancers18193140

NCT05542485	PLAsma Genomic Testing in Patients With Advanced Non-Small Cell Lung Cancer: The PLAN Study (PLAN)	Observational	NA	3	NSCLC	Advanced stage	97	CtDNA	Genetic	Detection	NA	KRAS, EGFR, ERBB2, BRAF, MET, ALK, ROS1, NTRK, RET	NA	No study results posted on ClinicalTrials.gov for this study (Unknown status)	Aug-2023	Dec-2025
NCT07057102	Multiple Omics Sequencing Technologies in Predicting the Efficacy and Monitoring the Recurrence of Non-Small Cell Lung Cancer	Observational	NA	1	NSCLC	Stage II Stage III Stage IV	40	CtDNA	NA	Cancer management	Sequencing	NA	NA	No study results posted on ClinicalTrials.gov for this study (Recruiting)	Jan-2025	Dec-2027
NCT05257967	CSF Analysis in EGFR Mutant Non-Small Cell Lung Cancer With Leptomeningeal Disease	Interventional	NA	1	NSCLC	NA	10	CtDNA	Genetic	NA	ddPCR NGS	EGFR	NA	No study results posted on ClinicalTrials.gov for this study (Recruiting)	Jul-2022	Dec-2026
NCT06301295	Feasibility of Targeted Bronchial Washing for Molecular Testing by Next Generation Sequencing in Early-stage Lung Cancer	Interventional	NA	1	Lung cancer	Early stage	50	NA	Genetic	NA	NGS	NA	NA	No study results posted on ClinicalTrials.gov for this study (No longer looking for participants)	May-2024	Dec-2026
NCT05221372	ProSpecTive sAmpling in dRiver muTation Pulmonary Oncology Patients on Tyrosine Kinase Inhibitors (START-TKI) (START-TKI)	Observational	NA	1	NSCLC	Advanced or metastatic stage	1300	NA	Genetic	Treatment	ddPCR NGS	NA	NA	No study results posted on ClinicalTrials.gov for this study (Recruiting)	Feb-2017	Jan-2031
NCT04968548	Determination and Validation of a Multi-analyte Assay for Lung Cancer Screening	Observational	NA	31	Lung cancer	Early stage	5000	NA	NA	Screening	NA	NA	NA	No study results posted on ClinicalTrials.gov for this study (No longer looking for participants)	Sep-2021	Dec-2025
NCT03317080	Dynamic Monitoring Circulating Tumor DNA in Surgical Patients With Lung Cancer (LUNGCA)	Observational	NA	1	Lung cancer	Stage I-III	400	CtDNA	NA	Cancer management	Second-generation sequencing	NA	NA	No study results posted on ClinicalTrials.gov for this study (Unknown status)	Sep-2017	Sep-2023
NCT05790460	Telehealth Based Synchronous Navigation to Improve Molecularly-Informed Care for Patients With Lung Cancer (TESTING)	Interventional	NA	1	NSCLC	Advanced or metastatic stage	55	NA	NA	Treatment	NA	NA	NA	No study results posted on ClinicalTrials.gov for this study (No longer looking for participants)	May-2023	Nov-2024

NCT06141005	Feasibility of Bronchial Washing Fluid for Molecular Testing With Next Generation Sequencing in Lung Cancer	Interventional	NA	1	Lung cancer	NA	65	NA	Genetic	Detection	NGS	NA	NA	No study results posted on ClinicalTrials.gov for this study (No longer looking for participants)	Nov-2023	Dec-2024
NCT06054854	Biobank for the Identification of Biomarkers in Lung Cancer (BIRD, Biomarkers in Respiratory Disease) (BIRD-NK)	Observational	NA	1	Lung cancer	Stage III-IV	3000	NA	NA	Diagnosis Prognosis Treatment	NA	NA	NA	No study results posted on ClinicalTrials.gov for this study (Recruiting)	Jun-2024	Oct-2033
NCT07291921	To Conduct Multi-omics Integrated Studies in Peripheral Blood, Such as Fragment Omics, Metabolomics and Epigenetics, and Establish Non-invasive Dynamic Follow-up Monitoring Programs During Perioperative and Postoperative Periods (Observational Study)	Observational	NA	1	Lung Neoplasms	Stage IB-IIIA	100	CfDNA	Genetic Epigenetic	Follow-up	NA	NA	NA	No study results posted on ClinicalTrials.gov for this study (Recruiting)	May-2025	Oct-2027
NCT04529915	Multicenter Clinical Research for Early Diagnosis of Lung Cancer Using Blood Plasma Derived Exosome	Observational	NA	1	Lung cancer	N0 or N1 Patients with T1mi, Tsi, T1a, T1b, T1c, T2a and T2b stage	470	Exosome	NA	Diagnosis	ELISA Western blotting Deep learning analysis	NA	NA	No study results posted on ClinicalTrials.gov for this study (Unknown status)	Apr-2020	Dec-2023
NCT05921474	Detection of Circulating Tumor DNA After Stereotactic Ablative Radiotherapy in Patients With Unbiopsied Lung Tumors (SABR-DETECT)	Observational	NA	1	NSCLC	Stage I-IIA	100	CtDNA	Genetic	Diagnosis	NA	NA	NA	No study results posted on ClinicalTrials.gov for this study (Unknown status)	Apr-2023	Dec-2023
NCT05020275	Resistance to the Tyrosine Kinase Inhibitor Osimertinib and Pharmacokinetics in Non-small Cell Lung Cancer (RESISTYR)	Observational	NA	4	NSCLC	Advanced stage	60	CtDNA	Genetic	Treatment	NA	EGFR	NA	No study results posted on ClinicalTrials.gov for this study (No longer looking for participants)	Dec-2021	Mar-2027
NCT03774758	Biomarkers for Risk Stratification in Lung Cancer	Observational	NA	3	NSCLC	Early stage	590	CtDNA	NA	Detection	Guardant	NA	NA	No study results posted on ClinicalTrials.gov for this study (Recruiting)	Dec-2017	Dec-2030

https://doi.org/10.3390/cancers18193140

NCT04976296	MRD Monitoring in Lung Cancer After Resection	Observational	NA	1	NSCLC	Stage I-IIIA	300	CtDNA	NA	Follow-up	Geneplus OncoMRD lung assay	NA	NA	No study results posted on ClinicalTrials.gov for this study (Recruiting)	Sep-2021	Dec-2027
NCT05587114	Comparision of Various Biomarkers Between Peripheral and Pulmonary Blood	Observational	NA	1	Lung cancer	NA	150	Exosomes	NA	NA	ELISA	NA	NA	No study results posted on ClinicalTrials.gov for this study (Unknown status)	Dec-2022	Dec-2025
NCT06234579	Longitudinal Assessment of Genomic Alterations and Clonal Evolution in ALK-positive NSCLC (Galileo Project) (GALILEO)	Observational	NA	1	NSCLC	Stage IV	108	CtDNA	Genetic	Treatment	NGS	ALK	NA	No study results posted on ClinicalTrials.gov for this study (Recruiting)	Jul-2021	Jul-2026
NCT06036563	Prospective Screening and Differentiating Common Cancers Using Peripheral Blood Cell-Free DNA Sequencing	Observational	NA	8	Lung cancer	NA	3200	CfDNA	NA	Screening	Sequencing	NA	NA	No study results posted on ClinicalTrials.gov for this study (Unknown status)	Sep-2023	Dec-2025
NCT05517083	Feasibility of Intratumoral Washing Fluid for Detecting EGFR Mutations in Lung Cancer	Interventional	NA	1	NSCLC	Stage IV	41	NA	Genetic	Detection	Cobas real-time PCR a ddPCR	EGFR	NA	No study results posted on ClinicalTrials.gov for this study (No longer looking for participants)	Apr-2022	Dec-2024
NCT05227261	Early Detection of Five Common Cancers Using the ctDNA Analysing Test (K-DETEK)	Observational	NA	1	Lung cancer	NA	9057	CtDNA	NA	Detection	NA	NA	NA	No study results posted on ClinicalTrials.gov for this study (No longer looking for participants)	Apr-2022	Apr-2024
NCT07060950	Analysis of Circulating Tumor Markers in Blood - ALCINA 5 (ALCINA 5)	Interventional	NA	1	Lung cancer NSCLC	NA	620	NA	NA	NA	NA	NA	NA	No study results posted on ClinicalTrials.gov for this study (Not yet recruiting)	Oct-2025	Jul-2035
NCT03833934	Study of Plasma NGS for Assessment, Characterization, Evaluation of Patients With ALK Resistance (SPACEWALK)	Observational	NA	1	NSCLC	NA	62	CfDNA	Genetic	Treatment	ALK	NGS	NA	Plasma testing successfully identified resistance mechanisms and additional genomic alterations that may help guide subsequent targeted therapy and monitor disease evolution.	Jan-2019	Dec-2023
NCT03512847	Predictive Gene Profiles and Dynamic Measurement of Treatment Response in Metastatic Non-small Cell Lung Cancer	Observational	NA	1	NSCLC	Metastatic stage	150	CtDNA	NA	Treatment	NA	NA	NA	No study results posted on ClinicalTrials.gov for this study (No longer looking for participants)	Mai-2018	Nov-2022

NCT04490564	Validation of Molecular Diagnostic Assays to Detect Cancer Biomarkers in Blood and Primary Tumor in HNSCC/ NSCLC/ Melanoma	Observational	NA	6	NSCLC	Metastatic stage	135	CTC CtDNA CtRNA	NA	Diagnosis	PD-L1	NA	NA	No study results posted on ClinicalTrials.gov for this study (No longer looking for participants)	Jun-2019	Sep-2022
NCT03360630	Anti-PD-1 Alone or Combined With Autologous Cell Therapy in Advanced NSCLC	Interventional	Phase I Phase II	1	NSCLC	Advanced stage	60	NA	Genetic	Treatment	PD-1	NA	NA	No study results posted on ClinicalTrials.gov for this study (No longer looking for participants)	Nov-2016	Jun-2023
NCT02597738	A Feasibility Study to Further the Development of Lung Cancer-based Precision Medicine	Interventional	NA	1	Lung Cancer	Mixed stages (I–IV)	330	ctDNA	Genetic	Treatment;	NGS	EGFR	NA	No study results posted on ClinicalTrials.gov for this study (Not recruiting, No longer looking for participants	Jan-2016	Dec-2028
NCT06270992	Oral Microbiome Diagnostics of Lung Cancer	Observational	NA	1	Lung Cancer	NA	676	NA	NA	Diagnosis; Screening	Deep learning	NA	NA	No study results posted on ClinicalTrials.gov for this study (Recruiting)	Nov-2022	May-2026
NCT04945317	ROSE for Improved Molecular Marker Testing Via EBUS	Interventional	NA	4	NSCLC	NA	340	NA	NA	Treatment	NA	NA	NA	No study results posted on ClinicalTrials.gov for this study (Enrolling by invitation)	May-2021	Dec-2026
NCT07410494	Biomarker-Guided Allogeneic Single-Target or Dual-Target CAR-NK Cell Therapy for Advanced Solid Tumors	Interventional	I-II	1	NSCLC	metastatic	85	ctDNA; CTC; Exosomes / Extracellular Vesicles	Genetic	Treatment	CTC	TROP2, MSLN (Mesothelin), CD276 (B7-H3), HER2 (ERBB2), EGFR, GD2, CLDN18.2 (Claudin18.2), GPC3, PSMA (FOLH1)	NA	No study results posted on ClinicalTrials.gov for this study (Recruiting)	Jun-2026	Dec-2028
NCT07370077	Research on Early Screening and Diagnosis of Pulmonary Nodules Based on Novel Non-invasive Technologies.	Observational	NA	1	Lung Cancer	Early-stage	1800	ctDNA	Genetic	Detection, Follow-up, Treatment,	NGS	EGFR, ALK, ROS1, BRAF, KRAS, MET, RET, NTRK	NA	No Study Results Posted on ClinicalTrials.gov for this Study(Recruiting)	Dec-2022	Jun-2026
NCT05623319	Pembrolizumab and Olaparib Treatment of Extensive Small Cell Lung Cancer (ES-SCLC)	Interventional	II	12	SCLC	Extensive stage	60	ctDNA	Genetic	Follow-up; Treatment; Prognosis;	NA	NA	NA	No Study Results Posted on ClinicalTrials.gov for this Study(Recruiting)	Mar-2023	Jul-2027

NCT04496674	Bispecific PSMAxCD3 Antibody CC-1 in Patients With Squamous Cell Carcinoma of the Lung	Interventional	I-II	2	NSCLC	Metastatic stage	3	NA	NA	Treatment	NA	NA	NA	No Study Results Posted on ClinicalTrials.gov for this Study(Terminated, premature termination of the trial due to bad recruitment, No longer looking for participants)	Feb-2022	May-2023
NCT05414123	A Therapy Treatment Response Trial in Patients With Leptomeningeal Metastases ((LM) Using CNSide	Observational	NA	4	NSCLC	Advanced stage metastatic stage	40	CTC	Genetic	Diagnosis, Follow-up, Treatment,	FISH,NGS	NA	NA	No Study Results Posted on ClinicalTrials.gov for this Study(Terminated , The sponsor can no longer financially support the trial., No longer looking for participants)	Sep-2022	Oct-2023
NCT06328621	Lung Cancer Risk Assessment and Etiology	Observational	NA	1	Lung Cancer	NA	109	NA	Genetic	Detection	NA	NA	NA	No Study Results Posted on ClinicalTrials.gov for this Study(Completed, No longer looking for participants)	Jul-2021	Mar-2026
NCT05366881	cfDNA Assay Prospective Observational Validation for Early Cancer Detection and Minimal Residual Disease	Observational	NA	17	Lung Cancer	NA	7000	cfDNA	Epigenetic	Detection, Screening, Follow-up, Treatment monitoring (MRD)	NGS	NA	NA	No Study Results Posted on ClinicalTrials.gov for this Study (Recruiting)	May-2022-	Mar-2027
NCT07670442	Epigenomic Profiling of Circulating Cell-Free DNA (cfDNA) to Characterize the Dynamic Evolution of Molecular Subtypes in Extensive-Stage Small Cell Lung Cancer During First-Line Chemoimmunotherapy	Observational	NA	NA	SCLC	NA	50	cfDNA	Epigenetic	Follow-up, Treatment monitoring, Prognosis	NA	NA	NA	No Study Results Posted on ClinicalTrials.gov for this Study (Not yet recruiting)	Jul-2026	Jul-2030
NCT05641870	Molecular Profiling and Dynamic Changes of ctDNA in Unresectable Locally Advanced NSCLC	Observational	NA	1	NSCLC	Stage III	80	ctDNA	Genetic	Follow-up, Treatment monitoring, Prognosis	NGS	NA	NA	No Study Results Posted on ClinicalTrials.gov for this Study (Recruiting)	Feb-2021	Dec-2023
NCT05458726	Correlation Between Efficacy of Osimertinib and EGFR T790M Status and Ratio Via ddPCR in NSCLC	Interventional	II	1	NSCLC	Stage IV	80	ctDNA)	Genetic	Treatment , Follow-up	ddPCR, NGS	EGFR	NA	No Study Results Posted on ClinicalTrials.gov for this Study(Recruiting)	Sep-2020	Oct-2022

NCT06391749	Clinical Validation of an MCED Test in Symptomatic Populations (K-ACCELERATE)	Observational	NA	1	Cancer Lung	all stages	1000	cfDNA	Epigenetic	Detection, Diagnosis, Screening y	SPOT-MAS, NGS	NA	NA	No Study Results Posted on ClinicalTrials.gov for this Study	Jul-2024	Jul-2026
NCT06963307	Novel Epigenetic Mapping in Cell-Free DNA for the Detection of Lung Cancer	Observational	NA	1	Lung cancer	all stages	300	cfDNA	Epigenetic	Detection, Diagnosis, Screening, Follow-up, Treatment monitoring, Prognosis	NA	NA	NA	No Study Results Posted on ClinicalTrials.gov for this Study (Recruiting)	May-2025	Nov-2026
NCT02633189	Study Comparing Bevacizumab + Erlotinib vs Erlotinib Alone as First Line Treatment of Patients With EGFR Mutated Advanced Non Squamous Non Small Cell Lung Cancer	Interventional	III	55	Lung cancer	Stage IIIB/IV	200	ctDNA	Genetic	Follow-up, Treatment	NA	EGFR	NA	No Study Results Posted on ClinicalTrials.gov for this Study (Active, not recruiting)	Apr-2016	Jul-2024
NCT06503042	A Study to Prospectively Validate Blood Markers Associated With Differences in Efficacy of Neoadjuvant Therapy for NSCLC	Observational	NA	NA	NSCLC	NA	100	ctDNA	NA	Treatment, Prognosis	NA	NA	NA	No Study Results Posted on ClinicalTrials.gov for this Study (Not yet recruiting)	Aug-2024	Aug-2026
NCT03755102	A Pilot Study of Dacomitinib With or Without Osimertinib for Patients With Metastatic EGFR Mutant Lung Cancers With Disease Progression on Osimertinib.	Interventional	Phase I	8	Lung Cancer	Stage IV	17	cfDNA	Genetic	Treatment, Follow-up	NA	EGFR	NA	No Study Results Posted on ClinicalTrials.gov for this Study (Completed, No longer looking for participants)	Nov-2018	Jul-2023
NCT06122077	Clinical Validation of Freenome Multiomics Blood Test for Lung Cancer Screening	Observational	NA	36	Lung Cancer	NA	20000	cfDNA	NA	Screening, Detection, Diagnosis	NA	NA	NA	No Study Results Posted on ClinicalTrials.gov for this Study (Recruiting)	Nov-2023	Jun-2027
NCT05873790	Minimal Residual Disease Dynamic Monitoring in First-Line Serplulimab Plus Chemotherapy in Treatment of Extensive Small Cell Lung Cancer: An Observational Study	Observational	NA	NA	SCLC	NA	30	ctDNA	Genetic	Follow-up, Treatment, Prognosis	NGS	NA	NA	No Study Results Posted on ClinicalTrials.gov for this Study (Unknown status, Not yet recruiting)	Jun-2023	Dec-2024

https://doi.org/10.3390/cancers18193140

NCT06181929	A Prospective Study for the Early Diagnosis of Lung Cancer in Korean Females	Interventional	NA	1	Lung Cancer	NA	200	ctDNA	NA	Screening, Detection, Diagnosis	NA	NA	NA	No Study Results Posted on ClinicalTrials.gov for this Study (Recruiting)	Jun-2024	May-2034
NCT06145750	Implementing Fragmentomics Into Real World Screening Interven-Tions to Evaluate Clinical Utility Among Individuals With Elevated Risk for Lung Cancer	Interventional	NA	3	Lung Cancer	NA	28	cfDNA	NA	Screening, Detection, Diagnosis	NA	NA	NA	No Study Results Posted on ClinicalTrials.gov for this Study (Completed, No longer looking for participants)	Oct-2023	Aug-2025
NCT04372732	Serum Autoantibodies in Predicting the Efficacy of Anti-PD-1 Treatment in Patients With Advanced NSCLC	Observational	NA	NA	NSCLC	Advanced stage	200	NA	NA	Treatment, Prognosis	NA	NA	NA	No Study Results Posted on ClinicalTrials.gov for this Study (Unknown status, Not yet recruiting)	Sep-2020	Aug-2022
NCT06167460	Analysis of Circulating Tumor DNA Dynamics to Predict and Monitor Response to TKI in Patients With Advanced NSCLC.	Observational	NA	1	NSCLC	Stage IIIB–IV	50	ctDNA	Genetic	Follow-up, Treatment , Prognosis y	K-Track™ ctDNA assay	NA	NA	No Study Results Posted on ClinicalTrials.gov for this Study (Recruiting)	Dec-2023	Dec-2025
NCT04636047	NGS-based Comprehensive Genomic ctDNA Panel in NSCLC With Immunotherapy	Observational	NA	1	NSCLC	NA	450	ctDNA	Genetic	Treatment, Follow-up, Prognosis	NGS	NA	NA	No Study Results Posted on ClinicalTrials.gov for this Study (Unknown status, Recruiting)	Nov-2020	Aug-2023
NCT07257471	Application Research of Methylation Markers in Early-stage Lung Cancer Patients Treated With Co-ablation System Therapy	Observational	NA	1	NSCLC	Early-stage	30	ctDNA	Epigenetic	Follow-up, Treatment, Prognosis	NA	SHOX2 and PTGER4	NA	No Study Results Posted on ClinicalTrials.gov for this Study (Recruiting)	Jun-2025	Jun-2028
NCT07106528	Organoid-guided vs Topotecan Therapy in Relapsed Extensive-Stage Small Cell Lung Cancer	Interventional	Phase II Phase III	NA	SCLC	NA	128	NA	NA	Treatment,	NA	NA	NA	No Study Results Posted on ClinicalTrials.gov for this Study (Not yet recruiting)	Aug-2025	Dec-2028
NCT07524114	Study of High-Precision Evaluation of Molecular ResiduaL Disease Through a PlatfOrm for Cancer TracKing and Interception (SHERLOCK)	Observational	NA	1	Lung Cancer	Early-stage	7000	ctDNA	Genetic	Detection, Follow-up, Prognosis	NGS	NA	NA	No Study Results Posted on ClinicalTrials.gov for this Study (Recruiting)	Mar-2026	Mar-2031

https://doi.org/10.3390/cancers18193140

NCT07558447	Extracellular Vesicles and Chemotherapy-Induced Peripheral Neuropathy	Interventional	NA	1	Lung Cancer	NA	120	Exosome	Genetic	Detection, Follow-up, Prognosis	NA	miRNAs	NA	No Study Results Posted on ClinicalTrials.gov for this Study (Recruiting)	Jan-2025	Sep-2026
NCT07070011	Small Extracellular Vesicle miRNAs as Predictive Biomarkers for Immunochemotherapy Efficacy in Extensive-stage Small Cell Lung Cancer	Observational	NA	1	Lung Cancer	NA	38	Exosome	Genetic	Treatment, Prognosis	NGS	miRNAs	NA	No Study Results Posted on ClinicalTrials.gov for this Study (Recruiting)	Jan-2023	Dec-2026
NCT04806412	Oncodrivers in Malignant Pleural Effusions Associated With Non-small Cell Lung Cancer: A Prospective Study.	Interventional	NA	1	NSCLC	Advanced	100	NA	Genetic	Diagnosis, Treatment	Immunohistochemistry (IHC)	EGFR, ALK, PD-L1	NA	No Study Results Posted on ClinicalTrials.gov for this Study (Recruiting)	Mar-2021	Dec-2028
NCT04957602	Evaluation of a Novel Microfluidic Device to Purify Metastatic Lung Cancer Patients CTC (Circulating Tumoral Cells)	Observational	NA	1	Lung Cancer	Stage IV	6	ctDNA, CTC	Genetic	Screening, Follow-up, Prognosis	NGS	NA	NA	No Study Results Posted on ClinicalTrials.gov for this Study (Terminated, No longer looking for participants)	May-2022	Sep-2022
NCT06321640	Study for the Multidimensional Analyses of Resistance and Toxicity to Immune- and Targeted-therapies.	Observational	NA	1	Lung Cancer	NA	265	ctDNA	Genetic	treatment	NA	NA	NA	No Study Results Posted on ClinicalTrials.gov for this Study (Unknown status, Recruiting)	Jul-2022	Dec-2025
NCT05451056	An Open-label, Phase 2trial of Sotorasib in KRAS G12C-mutant Non-small-cell Lung Cancer(NSCLC) Patients and a Translational Study to Find Acquired Resistance Mechanism to Sotorasib	Interventional	Phase II	1	NSCLC	NA	37	ctDNA	Genetic	Treatment, Follow-up,	NA	KRAS	NA	No Study Results Posted on ClinicalTrials.gov for this Study (Unknown status, Not yet recruiting)	Aug-2022	Feb-2026
NCT06816979	Lumbar Punctures for the Detection of ctDNA in the Cerebrospinal Fluid of Patients With Stage III and IV Non-Small Cell Lung Cancer	Interventional	NA	1	Lung Cancer	Stage III Stage IV	40	ctDNA, CTC	Genetic	Detection, Screening, Prognosis, Follow-up,	NGS	NA	NA	No Study Results Posted on ClinicalTrials.gov for this Study (Not yet recruiting)	Sep-2026	Jan-2028
NCT07564557	Septum-guided Segmentectomy for Deep Early-stage Lung Cancer (SGS2512)	Interventional	NA	1	Lung Cancer	stage IA	89	NA	NA	NA	NA	NA	NA	No Study Results Posted on ClinicalTrials.gov for this Study(Recruiting)	Apr-2026-	Apr-2031

https://doi.org/10.3390/cancers18193140

NCT05461430	Mass Response of Tumor Cells as a Biomarker for Rapid Therapy Guidance (TraveraRTGx)	Observational	NA	1	NSCLC	NA	72	NA	Genetic	Treatment,	TraveraRTGx	NA	NA	No Study Results Posted on ClinicalTrials.gov for this Study(Completed, No longer looking for participants)	Jul-2022	Mar-2026
NCT07004712	Efficacy and Biomarkers of the Second-line Treatment of Adebelizumab Combined With Irinotecan Liposome in Advanced Small Cell Lung Cancer	Interventional	Phase II	1	Lung Cancer	NA	46	ctDNA	Genetic	treatment	NA	NA	NA	No Study Results Posted on ClinicalTrials.gov for this Study(Not yet recruiting)	Jun-2025	Sep-2028
NCT07288034	Immunotherapy Biomarkers to Predict First-line PD(L)1-based Immunotherapy Response and Selection of Second-line Treatment in Stage IIIB-IV Non-small Cell Lung Cancer, IMMUNO-BIOMAP Trial	Interventional	Phase II	13	Lung Cancer	Stage IIIB–IV	535	ctDNA	Genetic	Treatment, Follow-up, Prognosis,	Multi-omics analysis	STK11, KEAP1 KRAS	NA	No Study Results Posted on ClinicalTrials.gov for this Study (Recruiting)	Apr-2026	Oct-2028
NCT04985357	Defining the Clinical Potential of Mass Response As a Biomarker for Patient Tumor Sensitivity to Drugs	Observational	NA	NA	Lung Cancer	NA	0	NA	NA	Treatment,	Travera	NA	NA	No Study Results Posted on ClinicalTrials.gov for this Study (Withdrawn)	Nov-2024	May-2030
NCT05411107	Oral Iloprost for the Prevention of Lung Cancer In Former Smokers	Interventional	Phase II	NA	Lung Cancer	Stage I-II	0	NA	NA	Screening; Treatment follow-up	NA	NA	NA	No Study Results Posted on ClinicalTrials.gov for this Study (Withdrawn)	Jun-2024	Jun-2027
NCT06247826	Mechanisms of Resistance to Amivantamab in Patients With NSCLC With EGFR Exon 20 Insertion	Interventional	NA	18	Lung Cancer	NA	30	ctDNA	Genetic	Follow-up; Treatment Prognosis	NGS	EGFR	NA	No Study Results Posted on ClinicalTrials.gov for this Study (Recruiting)	Jan-2024	Jul-2026

https://doi.org/10.3390/cancers18193140

NCT02769962	Trial of EP0057, a Nanoparticle Camptothecin With Olaparib in People With Relapsed/Refractory Small Cell Lung Cancer	Interventional	Phase I I Phase II	1	Lung Cancer	Metastatic stage	45	NA	NA	Treatment	NA	NA	NA	This study demonstrated that the combination of EP0057 and olaparib had a manageable safety profile and established the recommended phase II dose of EP0057 12 mg/m² every 2 weeks plus olaparib 250 mg twice daily. The study also showed preliminary antitumor activity in patients with relapsed/refractory small-cell lung cancer, while incorporating translational biomarker analyses to investigate genomic predictors of treatment response and resistance.	May-2016	Mar-2025
NCT06969781	MWA With or Without Furmonertinib for Early-Stage Non-Small Cell Lung Cancer	Interventional	NA	NA	Lung Cancer	Early-stage	120	NA	NA	Treatment	NA	NA	NA	No Study Results Posted on ClinicalTrials.gov for this Study (Not yet recruiting)	May-2025	Dec-2028
NCT07188480	The Role of DNA and RNA in NGS Analyses for Advaced Stage NSCLC Patients	Interventional	NA	1	NSCLC	NA	10	EV-DNA, EV-RNA	Genetic	Diagnostic; Detection; Treatment Follow-up	NGS	EGFR	NA	No Study Results Posted on ClinicalTrials.gov for this Study(Recruiting)	Jan-2025	Oct-2025
NCT06717243	Genomic and Methylation Markers in SCLC and LCNEC for Chemo-Immunotherapy Resistance Prediction (STRATUS)	Observational	NA	2	SCLC	NA	111	ctDNA, CTC	Genetic Epigenetic	Follow-up; Treatment; Prognosis	NGS	NA	NA	No Study Results Posted on ClinicalTrials.gov for this Study (Recruiting)	Feb-2025	Dec-2028
NCT06717295	The CCANED-CIPHER Study: Early Cancer Detection and Treatment Response Monitoring Using AI-Based Platelet and Immune Cell Transcriptomic Profiling	Observational	NA	4	Lung Cancer	Stage I-IV	6000	Platelet RNA + Immune cell RNA + Extracellular RNA	Genetic	Diagnostic, Detection, Treatment monitoring-Follow-up, Prognosis	NA	NA	NA	No Study Results Posted on ClinicalTrials.gov for this Study(Recruiting)	Oct-202	Aug-2028

NCT05697198	PRospective REgistry of Advanced Stage CancER (PREFER) Patients to Assess Prevalence of Actionable Biomarkers and Driver Mutations to Address Disparities in Precision Medicine	Observational	NA	8	Lung Cancer	Stage III-IV	1429	ctDNA / cfDNA	Genetic	Diagnostic; Detection; Treatment selection; Prognosis	NGS	NA	NA	No Study Results Posted on ClinicalTrials.gov for this Study (Completed, No longer looking for participants)	Apr-2021	Dec-2024
NCT07631624	Methylated Biomarkers of Smoking as a Selection Tool in Participants for Lung Cancer Screening	Interventional	NA	1	Lung Cancer	NA	1000	ctDNA	Genetic	Treatment / Prognosis /	NGS	KRAS, CDKN2A, SMAD4, TP53	NA	No Study Results Posted on ClinicalTrials.gov for this Study (Recruiting)	Oct-2025	Jan-2028
NCT05167851	The Safety and Efficacy of First-line Lazertinib and Locally Ablative Radiotherapy in Patients With Synchronous Oligometastatic EGFR-mutant Non-small Cell Lung Cancer	Interventional	II	1	Lung Cancer	Stage IV	68	ctDNA	Genetic	Treatment / Prognosis	NA	EGFR	NA	No Study Results Posted on ClinicalTrials.gov for this Study(Unknown status, Not yet recruiting)	Dec-2021	Dec-2025
NCT05998993	Study to Evaluate SBRT for EGFR Mutant NSCLC Patients Receiving Osimertinib (CULTRO)	Interventional	NA	1	Lung Cancer	Stage IIIB–IV	35	ctDNA	Genetic	Prognosis / Treatment	NGS	EGFR	NA	No Study Results Posted on ClinicalTrials.gov for this Study(Not yet recruiting)	Nov-2023	Nov-2028
NCT06910761	Photon Craniospinal Irradiation for the Treatment of Leptomeningeal Disease Secondary to Breast Cancer or Non-small Cell Lung Cancer	Interventional	II	2	NSCLC	Stage IV	35	cfDNA	NA	Prognosis /	NA	NA	NA	No Study Results Posted on ClinicalTrials.gov for this Study (Recruiting)	May-2025	Oct-2028
NCT05091190	Immunotherapy Clearance and Phenotype of Circulating Tumor Cells in Lung and Head and Neck Cancers	Interventional	NA	2	Lung Cancer	Stage I–III	75	CTC	NA	Treatment / Prognosis	NA	NA	NA	No Study Results Posted on ClinicalTrials.gov for this Study (Active, not recruiting, No longer looking for participants)	Oct-2021	Dec-2026
NCT04564079	An Observational Study to Evaluate the Clinical Utility of the Oncomine Precision Assay Within the Exactis Network	Observational	NA	6	Lung Cancer	Stage IIIB–IV	100	ctDNA	Genetic	Treatment prognosis	NGS	EGFR, ALK, ROS1, RET, BRAF, KRAS, MET, ERBB2	tissue and blood	No Study Results Posted on ClinicalTrials.gov for this Study(Completed, No longer looking for participants)	Jun-2021	Nov-2023
NCT05463796	InAdvance: Surveillance, Prevention, and Interception in a Population at Risk for Cancer	Observational	NA	1	Lung Cancer	NA	500	NA	Genetic	Screening / Detection / Prognosis /	NGS (WGS/WES)	NA	NA	No Study Results Posted on ClinicalTrials.gov for this Study(Recruiting)	Apr-2023	Mar2032

NCT05746325	Tumor Treating Fields for the Treatment of Leptomeningeal Metastases of the Spine in Patients With Breast or Lung Cancer	Interventional	NA	1	Lung Cancer	Stage IV	5	NA	NA	Treatment /	NA	NA	NA	No Study Results Posted on ClinicalTrials.gov for this Study(Recruiting)	Apr-2023	Mar-2027
NCT05944237	HTL0039732 in Participants With Advanced Solid Tumours	Interventional	Phase I I Phase II	5	Lung Cancer	Stage I/IIa	150	ctDNA	Genetic	Treatment	NGS	PIK3CA, ERBB2 (HER2), PI3K/AKT/mTOR pathway driver mutations	NA	No Study Results Posted on ClinicalTrials.gov for this Study(Recruiting)	Jul-2023	Jun-2027
NCT06694454	Neoadjuvant Inhaled Azacytidine With Platinum-Based Chemotherapy and Durvalumab (MEDI4736) - a Combined Epigenetic-Immunotherapy (AZA-AEGEAN) Regimen for Operable Early-Stage Non-Small Cell Lung Cancer (NSCLC	Interventional	Phase I I Phase II	1	NSCLC	Stage IB–IIIA	60	NA	Epigenetic	NA	NA	NA	NA	No Study Results Posted on ClinicalTrials.gov for this Study(Recruiting)	Jul-2026	Dec-2034
NCT05122806	Analysis of Biological Characteristics of Advanced ALK-rearranged NSCLC	Interventional	NA	46	NSCLC	Stage IIIB–IV	100	ctDNA	Genetic	Detection, Follow-up, Treatment, Prognosis	NGS, RNA-seq	ALK	NA	No Study Results Posted on ClinicalTrials.gov for this Study(Recruiting)	Sep-2021	Jun-2027
NCT06500481	Testing Proton Craniospinal Radiation Therapy Versus the Usual Radiation Therapy for Leptomeningeal Metastasis, RADIATE-LM Trial	Interventional	Phase III	58	Lung Cancer	Stage IV	115	NA	NA	Treatment	NA	NA	NA	No Study Results Posted on ClinicalTrials.gov for this Study(Recruiting)	Mar-2025	Jul-2028
NCT05200481	Study of Safety and Efficacy of Brigatinib Plus Chemotherapy or Brigatinib Only in Advanced ALK-Positive Lung Cancer (MASTERPROTOCOL ALK)	Interventional	Phase II	29	Lung Cancer	metastatic stage	110	ctDNA	Genetic	Detection, Treatment, Prognosis	NGS	ALK	NA	No Study Results Posted on ClinicalTrials.gov for this Study(Active, not recruiting, No longer looking for participants)	May-2022	Oct-2028
NCT07492121	Screening for Brain Metastases	Interventional	Phase II	1	Lung Cancer	Stage IV	180	cfDNA	NA	Screening, Detection,	NA	NA	NA	No Study Results Posted on ClinicalTrials.gov for this Study(Not yet recruiting)	Sep-2026	Jan-2030

NCT05257551	Tempus Small Cell Lung Cancer Observational Study (Sculptor)	Observational	NA	11	Lung Cancer	NA	200	ctDNA, CTC	Genetic and Epigenetic	Detection, Follow-up, Treatment, Prognosis	NGS	NA	NA	No Study Results Posted on ClinicalTrials.gov for this Study(Recruiting)	Jul-2022	Mar-2029
NCT04285671	Necitumumab and Trastuzumab in Combination With Osimertinib for the Treatment of Refractory Epidermal Growth Factor Receptor (EGFR)-Mutated Stage IV Non-small Cell Lung Cancer	Interventional	Phase I Phase II	1	Lung Cancer	Stage IV	30	NA	Genetic	Treatment	NA	EGFR	NA	No Study Results Posted on ClinicalTrials.gov for this Study(Active, not recruiting, No longer looking for participants	Jan-2021	Dec-2027
NCT05748093	Improving Osimertinib Exposure and Cost-effectiveness Using Pharmacokinetic Boosting With Cobicistat	Interventional	IV	1	NSCLC	NA	60	NA	Genetic	Treatment	NA	EGFR	NA	No Study Results Posted on ClinicalTrials.gov for this Study (Recruiting)	Apr-2024	Sep-2026
NCT06518057	Hippocampal Avoidance in Craniospinal Irradiation for the Treatment of Leptomeningeal Metastases From Breast Cancer or Non-small Cell Lung Cancer	Interventional	II	2	NSCLC	Stage IV	22	NA	NA	Treatment	NA	NA	NA	No Study Results Posted on ClinicalTrials.gov for this Study (Recruiting)	Mar-2025	Jul-2028
NCT05892237	CIETAI and Sequential Radiotherapy in Squamous Lung Cancer	Interventional	II	1	Lung Cancer	NA	50	ctDNA	Genetic	Follow-up, Treatment	IHC	PD-L1	NA	No Study Results Posted on ClinicalTrials.gov for this Study (Unknown status, Recruiting)	Jul-2023	Jun-2026
NCT05145244	Copenhagen Master Observational Trial	Observational	NA	1	Lung Cancer	Stage IIIB/IIIC/IV	2400	cfDNA,	Genetic	Follow-up, Treatment, Prognosis	WGS, cfDNA analysis	EGFR, ALK, ROS1	NA	No Study Results Posted on ClinicalTrials.gov for this Study (Recruiting)	Aug2021	Dec-2027
NCT07675967	Clonal Hematopoiesis Chemotherapy and Radiation Effects Study	Observational	NA	1	Lung Cancer	NA	5000	cfDNA	Genetic	Prognosis	NA	NA	NA	No Study Results Posted on ClinicalTrials.gov for this Study (Recruiting)	Apr-2025	Mar-2035

https://doi.org/10.3390/cancers18193140
